# The recruitment of TRiC chaperonin in rotavirus viroplasms correlates with virus replication

**DOI:** 10.1128/mbio.00499-24

**Published:** 2024-03-12

**Authors:** Janine Vetter, Guido Papa, Kurt Tobler, Javier M. Rodriguez, Manuel Kley, Michael Myers, Mahesa Wiesendanger, Elisabeth M. Schraner, Daniel Luque, Oscar R. Burrone, Cornel Fraefel, Catherine Eichwald

**Affiliations:** 1 Institute of Virology, University of Zurich, Zurich, Switzerland; 2 Molecular Immunology Lab, International Centre for Genetic Engineering and Biotechnology, Trieste, Italy; 3 Department of Structure of Macromolecules, Centro Nacional de Biotecnología/CSIC, Cantoblanco, Madrid, Spain; 4 Proteomics Lab, International Centre for Genetic Engineering and Biotechnology, Trieste, Italy; 5 Institute of Veterinary Anatomy, University of Zurich, Zurich, Switzerland; 6 School of Biomedical Sciences, The University of New South Wales, Sydney, New South Wales, Australia; 7 Electron Microscope Unit, Mark Wainwright Analytical Centre, The University of New South Wales, Sydney, New South Wales, Australia; Washington University in St. Louis, St Louis, Missouri, USA; College of Veterinary Medicine, Cornell University, Ithaca, New York, USA

**Keywords:** TRiC, rotavirus, viral replication, chaperones, double-stranded RNA virus, viroplasm, NSP5, VP2

## Abstract

Rotavirus (RV) replication takes place in the viroplasms, cytosolic inclusions that allow the synthesis of virus genome segments and their encapsidation in the core shell, followed by the addition of the second layer of the virion. The viroplasms are composed of several viral proteins, including NSP5, which serves as the main building block. Microtubules, lipid droplets, and miRNA-7 are among the host components recruited in viroplasms. We investigated the interaction between RV proteins and host components of the viroplasms by performing a pull-down assay of lysates from RV-infected cells expressing NSP5-BiolD2. Subsequent tandem mass spectrometry identified all eight subunits of the tailless complex polypeptide I ring complex (TRiC), a cellular chaperonin responsible for folding at least 10% of the cytosolic proteins. Our confirmed findings reveal that TRiC is brought into viroplasms and wraps around newly formed double-layered particles. Chemical inhibition of TRiC and silencing of its subunits drastically reduced virus progeny production. Through direct RNA sequencing, we show that TRiC is critical for RV replication by controlling dsRNA genome segment synthesis, particularly negative-sense single-stranded RNA. Importantly, cryo-electron microscopy analysis shows that TRiC inhibition results in defective virus particles lacking genome segments and polymerase complex (VP1/VP3). Moreover, TRiC associates with VP2 and NSP5 but not with VP1. Also, VP2 is shown to be essential for recruiting TRiC in viroplasms and preserving their globular morphology. This study highlights the essential role of TRiC in viroplasm formation and in facilitating virion assembly during the RV life cycle.

**IMPORTANCE:**

The replication of rotavirus takes place in cytosolic inclusions termed viroplasms. In these inclusions, the distinct 11 double-stranded RNA genome segments are co-packaged to complete a genome in newly generated virus particles. In this study, we show for the first time that the tailless complex polypeptide I ring complex (TRiC), a cellular chaperonin responsible for the folding of at least 10% of the cytosolic proteins, is a component of viroplasms and is required for the synthesis of the viral negative-sense single-stranded RNA. Specifically, TRiC associates with NSP5 and VP2, the cofactor involved in RNA replication. Our study adds a new component to the current model of rotavirus replication, where TRiC is recruited to viroplasms to assist replication.

## INTRODUCTION

Virus factories are compartmentalized inclusions made to assemble the replication factors to generate virus progeny. For example, rotavirus (RV), a multisegmented double-stranded RNA (dsRNA) virus and member of the Reoviridae family, replicates in the so-called viroplasms. These structures are composed of several RV proteins and ss- and dsRNA appearing as cytosolic membrane-less electron-dense inclusions when visualized at the electron microscope (1–3). Several essential processes for the RV life cycle take place in the viroplasms corresponding to the replication and packaging of the 11 dsRNA genome segments (gs) in newly formed VP2 icosahedral core shells. This process is assisted by replication intermediates composed of the RNA-dependent RNA polymerase (RdRp), VP1, and the helicase and guanylyl-methyltransferase VP3, which are found underneath each of the core-shell fivefold axis (4, 5). VP2 also acts as a cofactor of VP1, at least *in vitro*, for the dsRNA synthesis (6). The nonstructural proteins NSP5 and NSP2 take part in this replication step because they associate with VP1 in a mechanism that is still not completely elucidated (7–9). Subsequently, the filled core shells are coated by a second protein layer of VP6 trimers, forming double-layered particles (DLPs), which bud to the adjacent endoplasmic reticulum (ER) to acquire the outer coat. The triple-layered particles (TLPs) comprise glycoprotein VP7 and spike protein VP4, which can be found with transient lipid membranes in the ER surrounding the viroplasms (10). Moreover, when detected by fluorescence microscopy, the viroplasms appear as cytosolic globular inclusions that are homogeneously distributed in the cell at early times post-infection (~4 hpi). The main building block for viroplasm formation is NSP5 (11–13); specifically, its hyperphosphorylated form was shown to be essential for forming viroplasms (7, 14–16). Additionally, phosphorylated NSP2 is a requirement for viroplasm formation as well (9, 17). Interestingly, the co-expression of NSP5 with either NSP2 or VP2 is sufficient to build viroplasm-like structures (VLSs) (12, 18, 19), which are morphologically identical to viroplasms but lack virus replication components and hence are unable to produce virus progeny. The VLSs are excellent simplified tools for studying the complex viroplasm organization.

It has been demonstrated that viroplasms are highly dynamic, being able to coalesce between them and move to the juxtanuclear region of the cell at increasing times post-infection (20–22). Furthermore, despite not yet being well-defined, several host factors have been identified as necessary for viroplasm formation and maintenance (21, 23–26). On one side, the initiation process for viroplasm formation requires a scaffold of lipid droplets by incorporating perilipin-1 (27, 28). Furthermore, the host cytoskeleton, actin filaments and microtubules, plays a role in the formation, maintenance, and dynamics of the viroplasms (21, 29, 30). In this context, NSP2 octamers directly associate with microtubules to promote viroplasm coalescence (8, 21, 31–33). Moreover, VP2 plays a role in viroplasm dynamics by allowing their perinuclear motion (21). Finally, consistent with the above-described features, the viroplasms have been recently attributed to liquid-liquid phase-separated structures (34).

The RdRp (VP1) has a special feature common to all RdRp enzymes in the Reoviridae family (35–38). This feature consists of four channels connecting the catalytic cavity with the exterior to permit the template and NTP entry, and the transcript and template exit (39). This enzyme synthesizes mRNA or positive-sense single-stranded RNA [(+)ssRNA] using as a template the negative-sense single-stranded RNA [(−)ssRNA] obtained by unwinding the dsRNA genome segments via the C-terminal domains (40, 41). Also, *in vitro* experiments have shown that purified VP1 synthesizes dsRNA using (+)ssRNA as a template by recognizing the 3′ consensus sequence of each genome segment in a process strictly assisted by purified VP2 (6, 42). There is no *in vivo* evidence identifying this mechanism for the encapsidation of the newly generated genome segments.

The tailless complex polypeptide I ring complex (TRiC), eukaryotic group II chaperonin, assists in folding about 10% of all cytosolic proteins. It mainly favors the folding of newly translated proteins with complex beta-sheet topologies, such as actin and tubulin, and cell cycle regulators (43). TRiC has a continuously increasing list of client proteins that are involved in diverse cellular processes (44, 45). TRiC is organized as two back-to-back hetero-octameric rings having a barrel-shaped structure enclosing an ATP-dependent folding chamber. Each ring comprises eight paralog subunits (CCT1–CCT8) that adjust according to the specificity required for client proteins through differential recognition modes and differentiate rates of ATP binding and hydrolysis between the ring subunits (44, 46). Recently, TRiC activity has also been found to assist the folding of several viral proteins involved in various steps of the virus life cycle, such as entry (47), virus replication (48–54), virion assembly (55, 56), and virus particle release (57).

Our study shows that TRiC plays a crucial role in assisting RV replication by being recruited in the viroplasms. Inhibition of TRiC derives from a shortage of TLPs in the ER surrounding viroplasms and, most importantly, DLPs/TLPs lacking VP1/VP3 complex and dsRNA genome segments. Furthermore, we reveal that TRiC is associated with NSP5 and VP2 but not VP1. Also, VP2 is shown to be essential for recruiting TRiC in viroplasms and preserving their globular morphology.

## RESULTS

### Association of TRiC components to NSP5-BioID2

**Fig 1:**
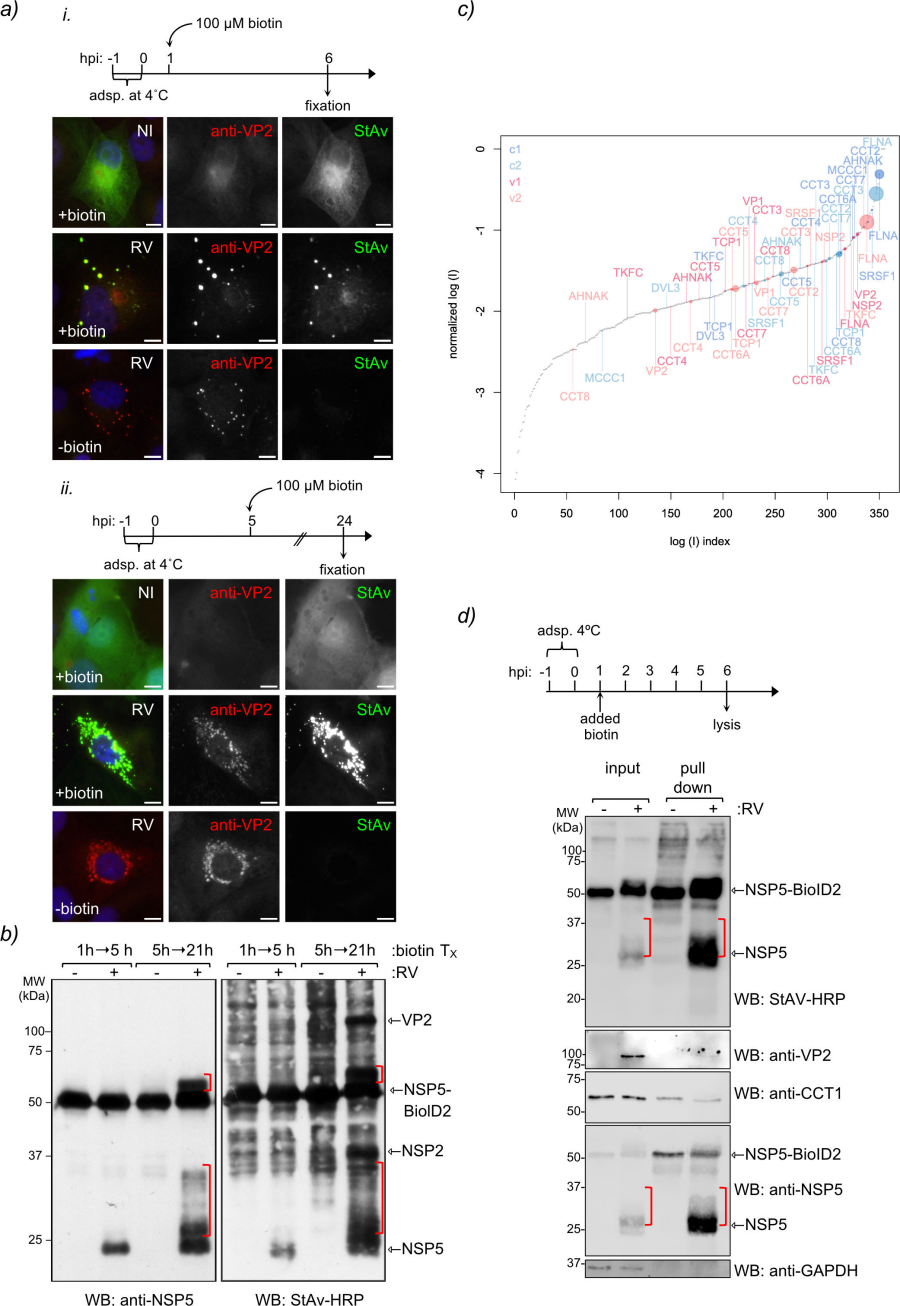
Tandem mass spectrometry analysis of pull-down proteins recruited to NSP5-BioID2 in viroplasms. (**a**) Immunofluorescence images of RV-infected MA/NSP5-BioID2 cells treated with biotin either at 1 hpi (upper panel) or 5 hpi (lower panel) and fixed at 6 or 24 hpi, respectively. Viroplasms were immunostained with anti-VP2 (Alexa 594, red), and NSP5-BioID2 was detected with streptavidin-dylight488 (green). Nuclei were stained with DAPI (blue). Non-infected (NI) control cells are indicated at the top row. The scale bar is 10 µm. (**b**) Western blot of non-infected and RV-infected MA/NSP5-BioID2 cell extracts pulled down with streptavidin agarose beads. The cells were treated with 100 µM biotin for the indicated time post-infection (*T*_*x*_). The membranes were incubated with anti-NSP5 (left) and streptavidin-HRP (right). Red brackets indicate the NSP5 hyperphosphorylation state. (**c**) Mass spectrometry of virus-infected and mock-infected cells. The log(*I*) values were normalized by subtracting the log(*I*) value of the NSP5 protein signal. The identified proteins were ranked according to the log of the normalized intensity signal from the mass spectrometry analysis. Proteins identified in the mock-infected samples were colored blue (c1 and c2), and proteins identified in virus-infected samples were colored red (v1 and v2). The sizes of the dots are inversely proportional to the log of the *e*-values from the mass spectrometry. (**d**) Western blot of streptavidin pull-down assay of non-infected and RV-infected NSP5-BioID2 cell lysates. As indicated in the upper scheme, the cells were treated at 1 hpi with biotin and lysed at 6 hpi. The input corresponds to 5% of cell lysates. The membrane was incubated with StAV-HRP and the indicated specific antibodies. Red brackets indicate the NSP5 hyperphosphorylation state.

To assess the interaction of RV proteins and host components in the viroplasms, we generated a stable MA104 cell line, MA/NSP5-BioID2, expressing NSP5 fused to BioID2, a promiscuous biotin ligase for the detection of protein-protein associations and proximate proteins in living cells (58). Similar to the NSP5-EGFP cell line (7, 20, 21), NSP5-BioID2 localizes and accumulates in viroplasms upon RV infection, as visualized by the co-localization of streptavidin (StAV)-dylight 488 with VP2, a marker for viroplasms, at 6 and 24 hpi (Fig. 1a). Next, we analyzed cell extracts of RV-infected MA/NSP5-BioID2 cells at either 5 or 21 hpi by immunoblotting (Fig. 1b). We found that most biotinylated proteins accumulate in cell extracts prepared at 21 hpi rather than at 5 hpi. Moreover, the bands consistent with the predicted molecular weight of NSP5, NSP2, and VP2 appeared at this specific time upon RV infection. The 21-hpi cell extracts were pulled down with StAv conjugated to magnetic beads and analyzed by label-free tandem mass spectrometry (MS/MS) (Fig. 1c; Table 1) that enabled the identification of 272 proteins. The distribution of non-infected and RV-infected samples is illustrated in the rank order plot in Fig. 1c. Consistent with previous publications (14, 20, 29, 59, 60) and validating our experimental procedure, NSP5-BioID2 pulled down RV proteins NSP5, NSP2, VP2, and VP1, which are recognized protein-protein interactors with NSP5. Interestingly, our study also identified the eight subunits (CCT1–CCT8) of the TRiC, a eukaryotic cytosolic ATP-dependent chaperonin that assists the folding of up to 10% of cytosolic proteins (45, 61). To confirm our result (Fig. 1d), we pulled down the RV-infected MA/NSP5-BioID2 cell extract treated with biotin (added at 1 hpi) and harvested at 6 hpi. As expected, NSP5-BioID2 biotinylated NSP5 and VP2, as denoted by immunoblotting using specific antibodies. We also identified the TRiC subunit CCT1 in both uninfected and RV-infected conditions, implicating association with NSP5-BioID2.

**TABLE 1:** List of ranked data obtained from mass spectrometry analysis of RV-infected MA-NSP5-BioID2 pulled-down extracts

Symbol^*a*^	Accession^*c*^	Control 1^*b*^		Control 2^*b*^		RV 1^*b*^		RV 2^*b*^		MW (kDa)^*f*^
		log(*e*)^*d*^	log(*I*)^*e*^	log(*e*)^*d*^	log(*I*)^*e*^	log(*e*)^*d*^	log(*I*)^*e*^	log(e)^*d*^	log(*I*)^*e*^	
ACACA	ENSP00000344789	−193.9	4.4	−219	8.65	−67.2	4.03	−135.2	8.25	269.8
FLNA	ENSP00000353467	−129	4.28	−204.4	8.73	−34.9	3.76	−206.9	8.67	279.8
NSP5	gi|210136690|	−59.3	4.59	−98.1	9.28	−113.4	4.99	−143.5	9.57	21.7
TCP1	ENSP00000317334	−2.4	2.78	−69.9	7.99	−14.5	3.24	−87	7.85	60.3
AHNAK	ENSP00000367263	−21	3.63	−66.3	7.74	−1.5	3.1	−7.5	7.19	628.7
CCT7	ENSP00000258091	-6	3.5	−38.3	7.93	−7.7	3.31	−37.1	7.97	59.3
CCT4	ENSP00000377958	−4.1	3.12	−37.6	7.59	−1.8	3.05	−40.8	7.69	57.9
SRSF1	ENSP00000258962	−20.2	3.84	−9.6	7.62	−23.8	3.6	−15.1	8.14	27.7
CCT8	ENSP00000373811	−7.3	3.29	−25.7	7.66	−12.1	3.42	−20.3	7.1	59.4
CCT5	ENSP00000280326	−1.1	3.07	−30.3	7.73	−3.5	3.14	−30.4	7.84	59.6
CCT3	ENSP00000295688	−8.6	3.18	−3.7	8.1	−6.9	3.34	−11.3	8.05	60.5
TKFC	ENSP00000378360	−1.5	2.76	−2.9	7.86	−1.4	2.9	−9.3	8.44	58.9
MCCC1	ENSP00000265594	-6	3.48	−13.7	7.04					
DVL3	ENSP00000316054	−2.1	2.76	-4	7.32					
VP2	gi|210136693|					−40.2	3.94	−53.9	7.58	102.7
NSP2	gi|210136684|					−32.7	3.9	−38.8	8.19	36.5
VP1	gi|210136695|					−9.9	3.33	−59.2	7.92	125.1

^*a*^ The proteins were ranked based on statistical confidence of the match.

^*b*^ Control and RV correspond to non-infected and RV-infected samples—illustrated data are from duplicated analysis.

^*c*^ Accession: the accession number for the matched protein.

^*d*^ Log (*e*): the statistical confidence of the match, expressed as log_10_ of the *e*-value.

^*e*^ Log (*I*): measures the overall intensity of the match.

^*f*^ The predicted molecular weight of the identified protein is indicated.

### TRiC localizes in viroplasms

**Fig 2:**
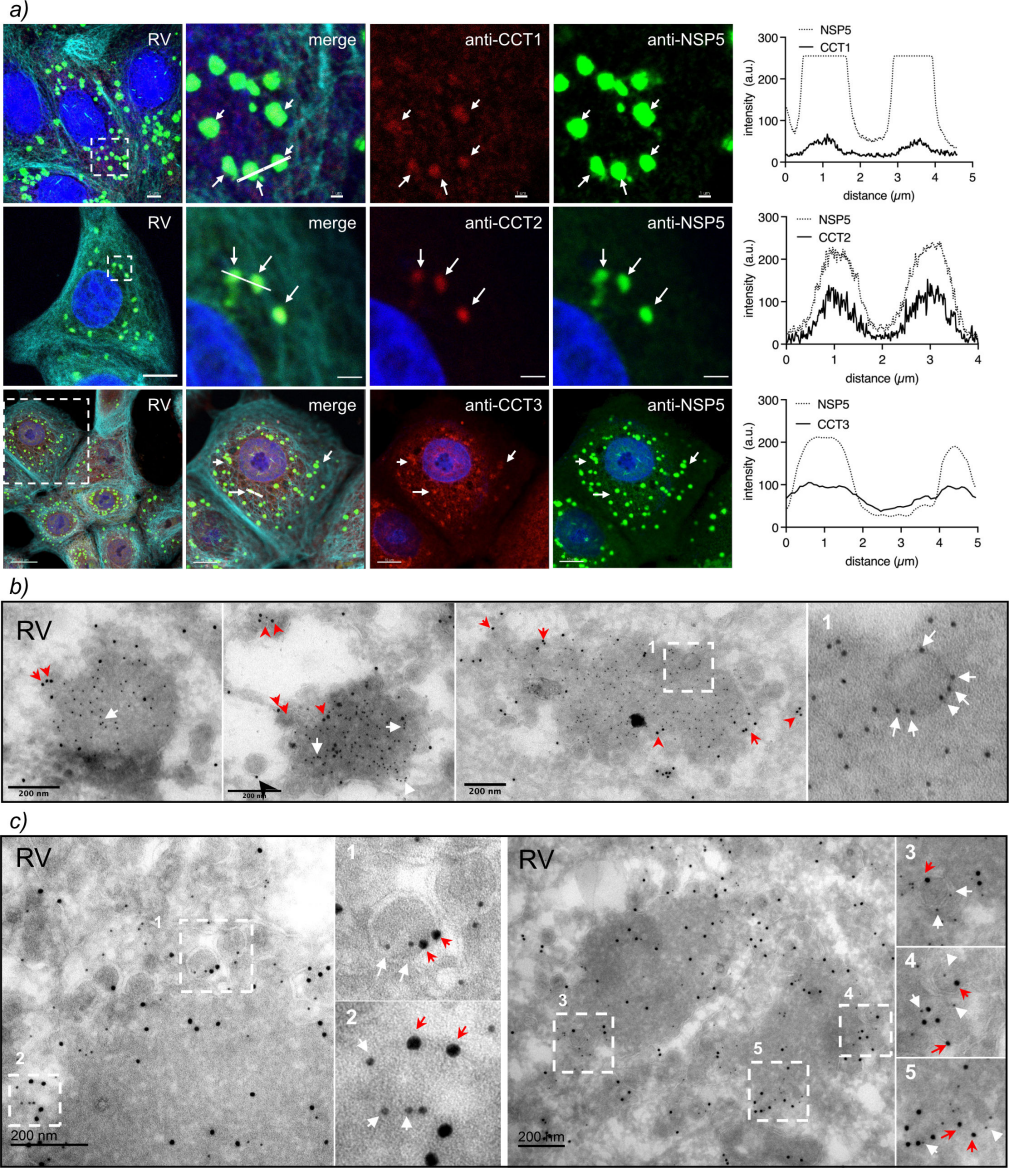
TRiC subunits localize in viroplasms surrounding virus particles. (**a**) Immunofluorescence of RV-infected cells immunostained at 6 hpi for the detection of viroplasms (anti-NSP5, Alexa 488, green), microtubules (anti-alpha tubulin, Alexa 647, cyan), and TRiC subunits CCT1, CCT2, and CCT3 (Alexa 594, red). Nuclei were stained with DAPI (blue). The white-dashed box represents the enlarged image at the right. White arrows point to the co-localization of viroplasms with TRiC subunits. The scale bar is 10 µm. The plots in the right column correspond to the co-localization profile of the linear region of interest of NSP5 with the TRiC subunit. Immune electron microscopy of viroplasm fixed at 6 hpi. The thin sections were co-immunostained with either anti-NSP5 conjugated to 6 nm gold (**b**) or anti-VP6 conjugated to 6 nm gold (**c**) followed by anti-CCT3 conjugated to 12 nm gold. The white-dashed open boxes correspond to enlarged indicated images. Red arrowheads and white arrows point to the localization of CCT3 and NSP5 or VP6 surrounding DLPs. The scale bar is 200 nm.

We next investigated whether TRiC localizes into viroplasms at 6 hpi to validate the mass spectrometry analysis. As visualized with immunofluorescence confocal microscopy (Fig. 2a), TRiC subunits CCT1, CCT2, and CCT3 colocalize with NSP5, a viroplasm marker, suggesting that TRiC is recruited to viroplasms. No crossreactivity was observed between TRiC-specific antibodies and RV antigens (Fig. S1). Next, we also analyzed viroplasms at high resolution using immune electron microscopy by co-immunostaining of anti-CCT3 (12 nm gold particles) with either anti-NSP5 (6 nm gold particles, Fig. 2b) or anti-VP6 (6 nm gold particles, Fig. 2c). Interestingly, CCT3, in addition to localizing in viroplasms, was found in some cases surrounding structures resembling DLPs by their morphology and size and because VP6 surrounds them. Interestingly, NSP5 and CCT3 are also found to encompass these globular structures. Like CCT3, also CCT2 colocalizes in viroplasms circumscribing DLPs (Fig. S2).

### Inhibition of TRiC hampers viroplasm formation and RV replication

**Fig 3:**
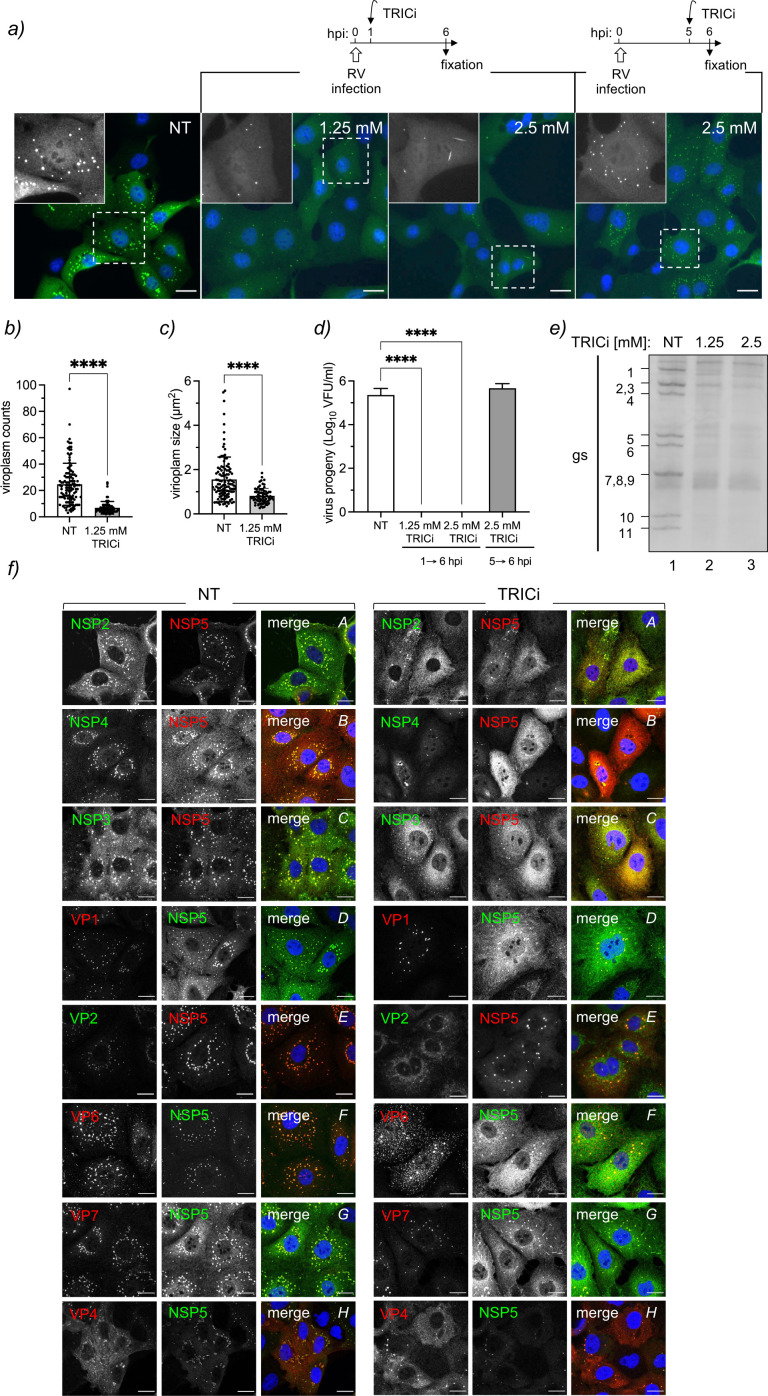
TRiC inhibition impairs viroplasm morphology and virus progeny. (**a**) Immunofluorescence micrograph of OSU-infected MA104 cells untreated or treated with 1.25 or 2.5 mM TRICi and fixed at 6 hpi. The compound inhibitor was added at 1 or 5 hpi as indicated. Cells were immunostained to detect viroplasms (anti-NSP5, green). Nuclei were stained with DAPI (blue). An enlarged image of a single cell is provided in black and white. The scale bar is 10 µm. Plots for the quantification of numbers (**b**) and size (**c**) of viroplasms per cell after TRICi treatment from 1 to 6 hpi. Data represent the mean ± SD. *n* > 50 cells; ****P < 0.0001. (**d**) Plot for virus progeny of RV-infected cells treated with TRICi during the indicated time post-infection. Data represent the mean ± SD of three independent experiments; ****P < 0.0001. (**e**) Electropherotype of RV gs extracted at 6 hpi from RV-infected cells non-treated (NT) and treated with 1.25 or 2.5 mM TRICi for 5 h before cell lysis. (**f**) Immunofluorescence micrograph of OSU-infected cells showing the distribution of RV proteins after treatment with 1.25 mM TRICi since 1 hpi. At 6 hpi, cells were fixed and immunostained to detect viroplasms [anti-NSP5; guinea pig polyclonal (green) or mouse monoclonal (red) antibodies] and the indicated RV protein (using specific antibodies for each of them). Nuclei were stained with DAPI (blue). The capital letters in the upper right corner correlate with the analyzed protein: A, NSP2; B, NSP4; C, NSP3; D, VP1; E, VP2; F, VP6; G, VP7; and H, VP4. Each panel shows untreated (NT, left panel) and 1.25 mM TRICi-treated (TRICi, right panel) samples. The scale bar is 10 µm.

We used a recently described chemical TRiC inhibitor (62) (PubChem CID: 658022) corresponding to 2-[(4- chloro-2λ4,1,3-benzothiadiazol-5-yl)oxy]acetic acid, shortly named TRICi, to investigate the role of TRiC chaperonin in the RV life cycle. TRICi was validated in MA104 cells for its ability to halt the onset of mitosis by impairing Cdc20 expression (Fig. S3a through e), a well-described protein dependent on TRiC for folding (63). When inspecting viroplasms at 6 hpi, a time frame for the visualization of well-formed viroplasms (21), RV-infected cells treated at early time post-infection (1 hpi) with TRICi showed significantly reduced numbers and size of viroplasms (Fig. 3a through c). In contrast, the addition of TRICi at 5 hpi showed no changes in the morphology and number of viroplasms compared to untreated samples (Fig. 3a). The TRICi treatment was not cytotoxic at the concentrations used in our experiments (Fig. S3f). Notably, the impairment in viroplasm formation after TRiC inhibition was observed for at least three RV strains, including porcine strain OSU and simian strains SA11 and RRV (Fig. S4a and b). The virus progeny was largely impaired (>5 log) when TRICi was added at 1 hpi at both tested concentrations but not when added at 5 hpi (Fig. 3d). Similarly, silencing either CCT3 or CCT2 subunits significantly reduced virus progeny (Fig. S4c through f). Consistent with the lack of virus progeny, we observed a complete depletion of the RV dsRNA genome segments (Fig. 3e) after TRICi treatment (lanes 2 and 3) when compared to untreated cells (lane 1).

Additionally, the effect of TRICi on viroplasms was partially reversible when added at 1 hpi and washed out at 2, 3, 4, or 5 hpi (Fig. S5a through e), followed by analysis at 6 hpi. Large viroplasms recovered even when removing the compound at 4 hpi, reaching the same levels as untreated samples. Meanwhile, the number of small viroplasms did not recover even after only 1 h of treatment with TRICi, remaining in large numbers distributed in the cytosol of the infected cells. Besides, the expression of diverse RV proteins was recovered after removing TRICi (Fig. S5f), which collectively suggests a delay in the coalescence of viroplasms because of a lack of RV protein supplies required for building the inclusions. Additionally, the distribution of diverse RV proteins was compared in untreated and TRICi-treated conditions by immunofluorescence at 6 hpi. As observed in Fig. 3f, RV viroplasm proteins NSP5, NSP2, VP2, and VP6 delocalized from the viral factories and dispersed throughout the cytosol, while VP1, NSP4, VP7, and VP4 did not show perceptible changes from their characteristic distribution. Also, NSP3 showed dramatic changes with a dispersed distribution.

### VP2-induced VLSs are impaired by inhibition of TRiC

**Fig 4:**
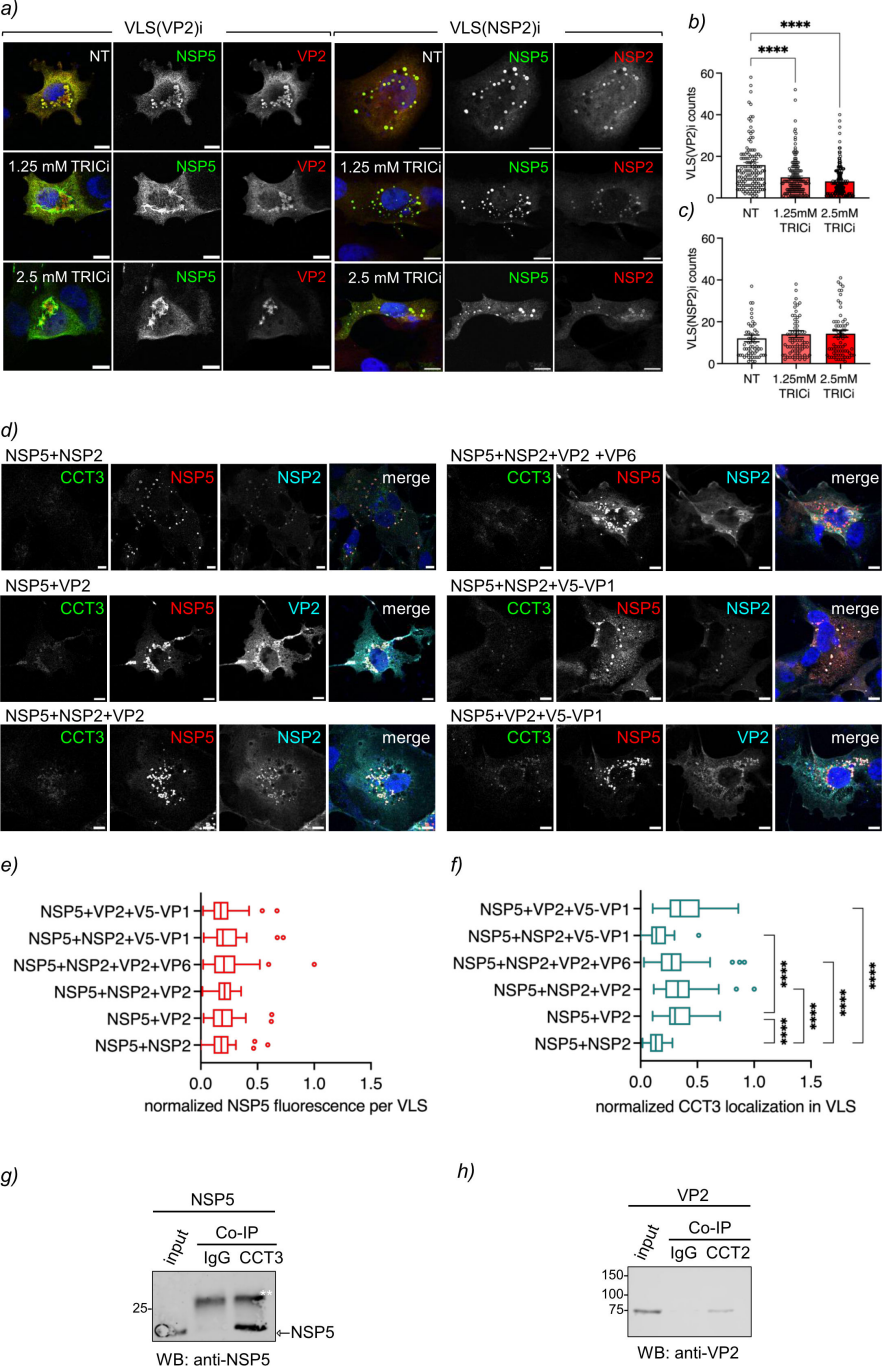
VP2 and NSP5 associate with TRiC. (**a**) Immunofluorescence of VLS induced with VP2 (left panel) or NSP2 (right panel) untreated (top row) or treated with TRICi (middle and bottom rows). The samples were fixed at 16 hpt and immunostained with specific antibodies for the visualization of NSP5 (green, Alexa 488), VP2 (red, Alexa 594), and NSP2 (red, Alexa 594). Nuclei were stained with DAPI (blue). Scale bar is 10 µm. Quantification of VLS plots induced by VP2 (**b**) or NSP2 (**c**) untreated or treated with TRICi at diverse concentrations. The data correspond to the mean ± SEM of >50 cells per experimental point. Welch-ANOVA where ****P < 0.0001. (**d**) Immunofluorescence images of VLSs composed of the indicated RV proteins. At 16 hpt, the cells were fixed and immunostained to detect CCT3 (anti-CCT3, Alexa 488, green), VLS (anti-NSP5, Alexa 594, red), and VP2 (anti-VP2, Alexa 647, cyan) or NSP2 (anti-NSP2, Alexa 647, cyan). Nuclei were stained with DAPI (blue). Scale bar is 10 µm. Plots for quantifying NSP5 (**e**) and CCT3 (**f**) localization in VLSs composed of the indicated RV proteins. The data means were compared using the Tukey method where *P < 0.05 and ****P < 0.0001. Immunoblotting of anti-TRiC immunoprecipitated from BHK/T7 cell lysates expressing NSP5 (**g**) and VP2 (**h**). The membranes were incubated with the indicated antibodies. The input corresponds to 5% of crude cell extract. IgG corresponds to immunoprecipitation with isotype control antibody. ** points to the light chain immunoglobulin.

As the assembly of viroplasms was compromised upon inhibition of TRiC, we wondered whether the formation of viroplasm-like structures was also affected. VLSs are built by the co-expression of NSP5 with either NSP2 or VP2. Indeed (Fig. 4a, left panel), the TRICi-treated VLS(VP2)i formed irregular filamentous structures that are significantly reduced in number (Fig. 4b) compared to untreated conditions. In contrast, VLSs induced by NSP2 [VLS(NSP2)i)] were undisturbed upon TRICi treatment in both morphology and number (Fig. 4a, right panel, and 4c). The VLS structures formed by co-expression of NSP5 and VP2 without TRiC inhibitor differ in form and localization from those formed by NSP2 and NSP5, consistent with the ability of VP2 to drive VLS to the perinuclear region (21), with apparent NSP5 protein diffusely distributed in the cytoplasm. As expected (7), the NSP5 hyperphosphorylation triggered by either VP2 or NSP2 was not affected upon TRICi treatment (Fig. S6a).

We also investigated the recruitment of TRiC to VLSs induced by the co-expression of NSP5 with VP2 or NSP2 and supplemented with VP6 or V5-VP1 (Fig. 4d; Fig. S6b). The diverse VLSs were monitored for the recruitment of the TRiC subunit CCT3 by confocal immunofluorescence, followed by quantification of the accumulation of the CCT3 signal in VLSs. All the combinations of assembled VLSs showed a similar morphology based on a homogenous NSP5 signal, a common marker for VLSs (Fig. 4e). We noticed that CCT3 localization in VLSs was enhanced in VLSs containing VP2 but not those with NSP2 (Fig. 4f). Similarly, VLSs containing additional VP6 or V5-VP1 did not improve CCT3 accumulation in VLSs, suggesting no role of these proteins in the recruitment of TRiC. Notably, CCT3 showed a basal accumulation in VLSs composed of NSP5 and NSP2, consistent with the ability of NSP5 to associate with TRiC. Other TRiC subunits, such as CCT1 and CCT2, were also localized in VLSs containing NSP5 and either NSP2 or VP2 (Fig. S6c). These results suggest that although both VP2 and NSP5 are associated with TRiC, VP2 is largely responsible for recruiting TRiC in the viroplasms. In this context, we investigated the association of NSP5 and VP2 with TRiC by co-immunoprecipitation using specific antibodies targeting TRiC subunits. For this purpose, NSP5 (Fig. 4g) or VP2 (Fig. 4h) expressed BHK/T7 cells were co-immunoprecipitated with TRiC and detected by immunoblotting using specific antibodies targeting these proteins. These results suggest that VP2 and NSP5 are associated with TRiC.

### Synthesis of (−)ssRNA is reliant on TRiC

**Fig 5:**
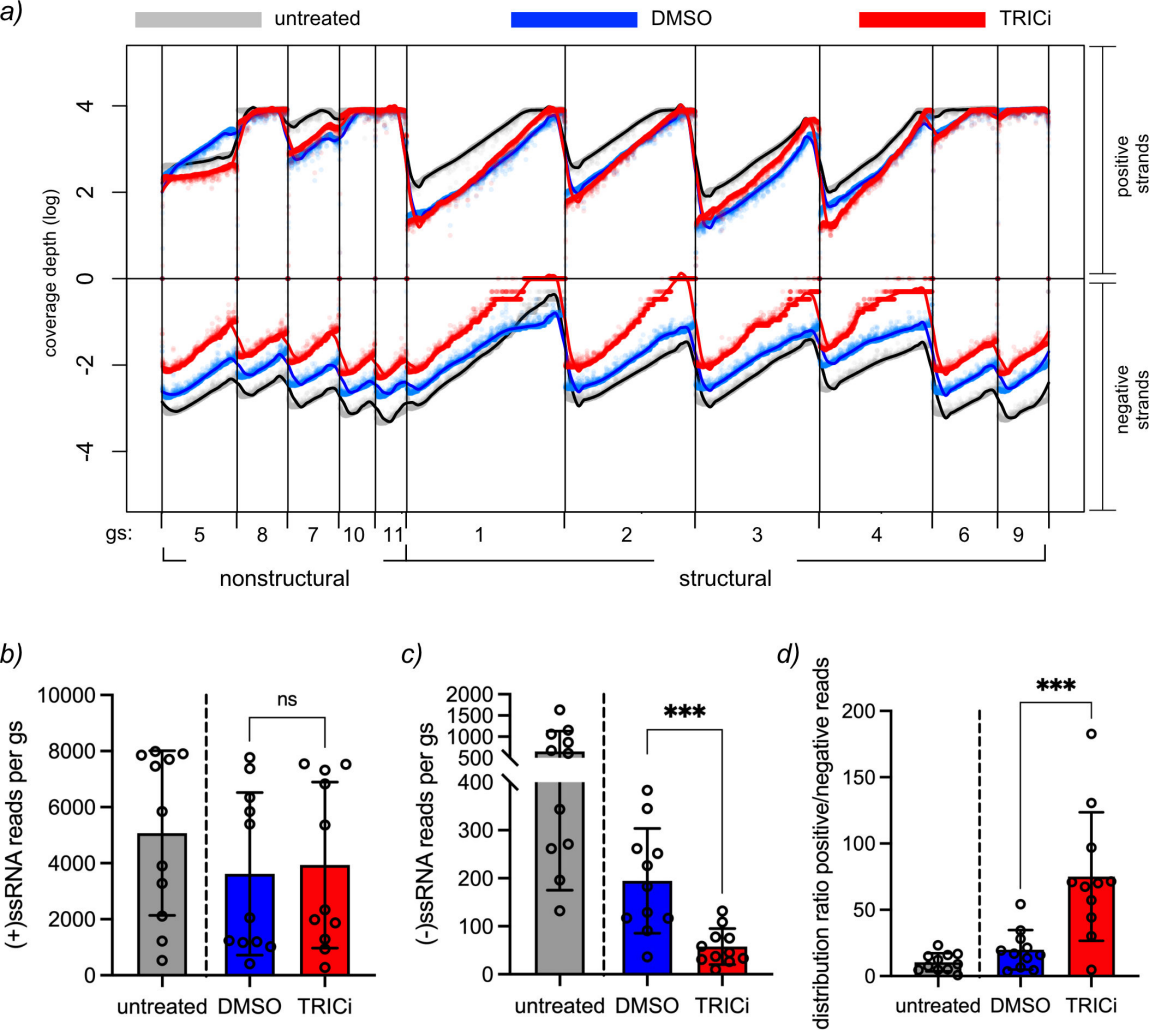
Rotavirus negative-strand synthesis is hampered upon inhibition of TRiC. (**a**) ONT for direct sequencing of rotavirus positive- and negative-sense RNA of the 11 genome segments from RV-infected cells at 6 hpi, untreated (gray point and black lines) or treated with compound carrier (DMSO, blue lines) or 2.5 mM TRICi (red lines). The chemical compound was added at 1 hpi until cell lysis. The plot indicates the depth of the sequence coverage in the logarithmic scale of the positive- (top) and negative (bottom)-sense RNA for the 11 RV genome segments, where nonstructural and structural proteins are indicated. While the experiment with untreated cells corresponds to a methodologic pilot experiment performed separately, the DMSO- and TRICi-treated RV-infected cell samples were prepared simultaneously and, consequently, compared statistically. Plot for the distribution of the positive (**b**) and negative (**c**) RNA reads per genome segment from untreated, DMSO-, and TRICi-treated RV-infected cells. (**d**) Plot comparing the ratio between positive and negative RNA sequence reads of RV-infected cell extracts untreated or treated with DMSO or TRICi. RM one-way ANOVA was performed between samples where ***P > 0.001.

Since dsRNA synthesis is not detected, but indeed RV proteins are expressed, it suggests a block in the synthesis of the (−)ssRNA. To address this possibility, we established a method for direct RNA sequencing of both positive- and negative-sense RNA strands from the 11 RV genome segments using MinION Oxford nanopore technology (ONT) (64). For this purpose, we designed specific reverse transcriptase adapters (RTA) that anneal to the 3′ ends of both positive- and negative-sense RNA of the 11 genome segments of porcine RV strain OSU (Table S1). Total RNA from OSU-infected cells at 6 hpi was harvested and sequenced via MinION to determine the effectiveness of the modified adapter. The sequence runs covered 100% of the 11 genome segments of both positive- and negative-sense RNA (Fig. 5a, gray dots and black lines). The average coverage depth for the positive-sense RNA ranked from 7,987.09 for gs 8 (NSP2) to 528.93 for gs 5 (NSP1), while that of the negative-sense RNA ranked from 1,632.10 for gs 11 (NSP5) to 132.48 for gs 4 (VP4) (Table 2). The average read level of accuracy of the 11 genome segments was 91.28% ± 0.46% and 90.05% ± 0.58% for the positive- and negative-sense RNA, respectively. The sequence coverage corresponded to 99.47% ± 0.32% for the positive-sense RNA and 99.28% ± 0.41% for the negative-sense RNA, following the consensus sequence of the 11 OSU genome segments. The distribution of positive- and negative-sense RNA read lengths (Fig. 5b and c, untreated) corresponds well to the expected length of each respective segment. A ratio distribution was determined between the positive and negative sense RNA reads for each genome segment with a mean value of 10.42 (Fig. 5d, untreated).

**TABLE 2:** ONT average coverage depth for (+) and (−) ssRNAs isolated from cells infected with porcine OSU strain

RNA sequence^*a*^	Average coverage depth (reads/nucleotide)	
	Positive strand	Negative strand
gs 1 (VP1)	3,282.45	196.16
gs 2 (VP2)	3,901.18	261.65
gs 3 (VP3)	1,223.30	271.19
gs 4 (VP4)	2,109.55	132.48
gs 5 (NSP1)	528.93	671.66
gs 6 (VP6)	7,705.13	864.61
gs 7 (NSP3)	7,465.08	607.89
gs 8 (NSP2)	7,987.08	343.86
gs 9 (VP7)	5,845.46	1,154.66
gs 10 (NSP4)	7,906.21	1,060.12
gs 11 (NSP5)	7,858.12	1,632.10
^*b*^GAPDH	4.12	0.00

^*a*^ Rotavirus porcine strain OSU.

^*b*^ Sequence covering GAPDH of *Macaca fascicularis*.

In the next experiment, we sequenced both positive- and negative-sense RNA strands of total RNA samples from RV-infected cells treated with either DMSO (compound carrier) or TRICi at 6 hpi and sequenced. Meanwhile, the sample from DMSO-treated cells showed complete coverage for both positive- and negative-sense RNA (Fig. 5a, blue lines; Table 3). However, the samples from TRICi-treated cells, although showing similar depth coverage for all the 11 segments as in the DMSO samples, denoted a highly reduced coverage for the 11 negative-sense RNA strands (Fig. 5a, red lines). Even if the distribution of the 11 genome segments for positive-sense RNA reads (Fig. 5b) was comparable between the two samples, it was highly diverse for the negative-sense RNA (Fig. 5c). In fact, the mean ratio of positive- or negative-sense RNA reads (Fig. 5d) for DMSO-treated cells is significantly lower than the TRICi-treated cells, suggesting a substantial reduction of (−)ssRNA synthesis.

**TABLE 3:** ONT average coverage depth for (+) and (−)ssRNAs isolated from OSU-infected cells untreated or treated with TRICi

RNA sequence^*a*^	Average coverage depth (reads/nucleotide)			
	DMSO		TRICi	
	Positive strand	Negative strand	Positive strand	Negative strand
gs 1 (VP1)	1,234.23	90.94	1,874.40	26.46
gs 2 (VP2)	2,055.77	128.91	2,340.52	33.37
gs 3 (VP3)	414.28	117.13	936.93	31.11
gs 4 (VP4)	1,021.22	36.40	1,283.85	9.83
gs 5 (NSP1)	1,167.30	261.97	275.04	58.00
gs 6 (VP6)	5,392.04	251.83	5,365.27	75.08
gs 7 (NSP3)	1,195.27	183.01	1,986.40	44.96
gs 8 (NSP2)	6,346.81	116.86	6,825.37	37.34
gs 9 (VP7)	7,771.91	226.33	7,526.26	77.71
gs 10 (NSP4)	5,846.58	345.27	7,324.93	108.73
gs 11 (NSP5)	7,377.46	383.15	7,548.64	131.79
^*b*^GAPDH	7.47	0.00	4.33	0.00

^*a*^ MA104 infected with porcine strain OSU.

^*b*^ Sequence covering GAPDH of *Macaca fascicularis*.

### TRiC inhibition produces empty DLP/TLPs

**Fig 6:**
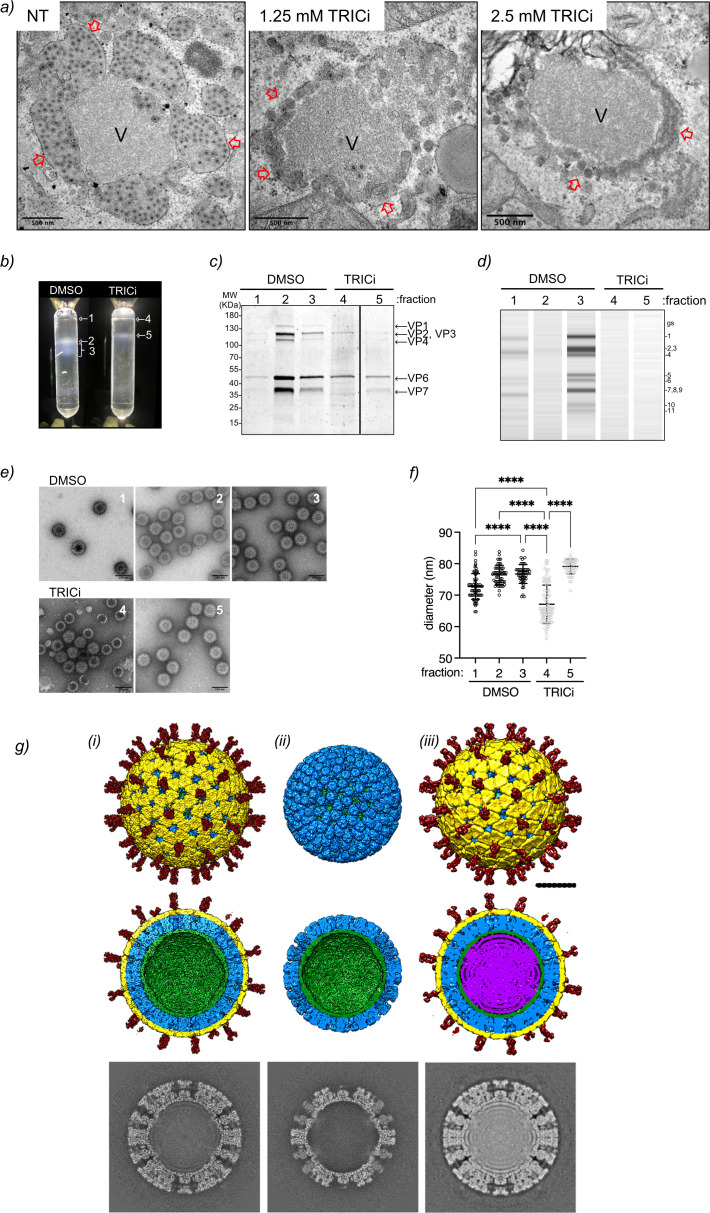
Inhibition of TRiC leads to empty DLPs. (**a**) High-definition electron microscopy of OSU-infected MA104 cells untreated and treated with TRICi at the indicated concentrations. The inhibitor was added at 1 hpi, and the samples were fixed at 6 hpi. The red, open arrowheads point to the endoplasmic reticulum surrounding viroplasms. The scale bar is 500 nm. (**b**) Image of purified OSU subviral particles with isopycnic cesium chloride gradient of infected cells untreated or treated at 1 hpi with 2.5 mM TRICi. The subviral particles were extracted at 8 hpi. The arrows point to the collected fractions. (**c**) Coomassie blue staining of subviral particles found in the indicated fractions. The arrows point to the corresponding structural proteins. (**d**) Analysis of dsRNA genome segments extracted from subviral particles of the indicated CsCl gradient fractions. Samples were detected with TapeStation Agilent using genomic DNAScreen Tape. (**e**) Negative staining of purified subviral particles from the indicated fractions of the CsCl gradient. (**f**) Plot corresponding to the size mean ± SD of the subviral particles fractions of CsCl gradient. One-way ANOVA, ****P-value < 0.001. (**g**) Cryo-EM structures of TLP and DLP derived from TRICi-treated cells. Cryo-EM 3D reconstructions of TRICi TLP (i), TRICi DLP (ii)*,* and control TLP [iii, EMD-2574 (67)]. Surface-shaded representation of the outer (top row) and inner (middle row) surfaces viewed along an icosahedral twofold axis. The surfaces are radially color-coded to represent VP5*/VP8* spikes (red), VP7 (yellow), VP6 (blue), VP2 (green), and VP1/genome (purple). The lower row represents 2.74 Å thick central sections of the maps. The scale bar is 250 Å.

To investigate in detail the decrease in both dsRNA genome segments and virus progeny associated with TRiC inhibition, we examined the viroplasms by high-resolution electron microscopy at 6 hpi after treatment with TRICi from 1 hpi. We observed that both untreated and TRICi-treated viroplasms were electron dense (Fig. 6a). As expected, the ER surrounding the viroplasms in the untreated sample was filled with TLPs at diverse stages of maturation. Simultaneously, TRICi-treated samples showed almost empty ER with a few immature virus particles. We then analyzed the virus particles isolated at 8 hpi from cells treated with TRICi at 1 hpi or untreated and separated by CsCl density gradient centrifugation to gain insights into virion structure and composition (Fig. 6b). In contrast to the three fractions observed from untreated samples (fractions 1–3), the TRiCi-treated samples presented only two fractions (4, 5). These two fractions, despite containing the TLP proteins (VP1, VP2, VP3, VP4, VP6, and VP7), lack the dsRNA genome (Fig. 6c and d; Table S2). As previously described (65, 66), fractions 2 and 3 corresponded mainly to empty TLPs and dsRNA-filled DLP/TLPs, respectively, as denoted by their protein and genome content (Fig. 6c and d). The low-density and less abundant fraction 1 contained all components of TLPs (VP1, VP2, VP3, VP4, VP6, and VP7), including the dsRNA genome segments and likely corresponding to unassembled subviral particles. Identical dilutions of the diverse fractions were also analyzed by negative staining electron microscopy (Fig. 6e), which showed a high abundance of subviral particles in fraction 4 compared to equivalent density fraction 1. When determining the size of particles (Fig. 6f), fraction 4 corresponded to a mixed population of particles with a diameter of 75 and 60 nm, which is consistent with the size of TLPs and DLPs, respectively.

To further characterize the particles isolated from TRICi-treated cells, we subjected fraction 4 to cryo-electron microscopy (cryo-EM). The examination of the cryo-EM images and subsequent 3D classification revealed that approximately one-third of the particles corresponded to TLPs, with distinct visualization of VP2, VP6, and VP7, along with conspicuous protruding VP5*/VP8* spikes (Fig. 6g(i)). The remaining two-thirds were identified as DLPs, exhibiting detectable densities attributed to VP2 and VP6 (Fig. 6g(ii)). Notably, neither TLP nor DLP 3D reconstructions displayed discernible density within the inner core that could be associated with the polymerase complex or the dsRNA genome. Comparative analysis of previously published reconstructions of RV TLPs (Fig. 6g(iii)), EMD-2574 (67), showcased a heightened density level in the internal radius of the particles, indicative of the presence of the genome and the VP1 + VP3 viral proteins. This stark contrast emphasizes the distinct structural features between TRICi-treated particles and their counterparts without the compound. This outcome suggests a preponderance of DLPs over TLPs, with an overall accumulation of empty particles lacking the virus genome and RdRp VP1 upon TRiC inhibition.

## DISCUSSION

The role of TRiC in folding the mammalian orthoreovirus (MRV) σ3, a member of the Reoviridae family, has been recently demonstrated to be essential for virus capsid assembly (55, 68). Using tandem mass spectrometry, we identified all TRiC subunits in RV viroplasms and found that TRiC plays an essential role in the RV life cycle, specifically in the synthesis of the dsRNA genome segments. Moreover, we provide evidence that the inhibition of TRiC results in fewer viroplasms, a shortage of TLPs in the ER, and defective TLP/DLPs lacking encapsidated genome segments and polymerase complex composed of VP1 and VP3 (PC). Our findings indicate that the inhibition of TRiC impairs the synthesis of (−)ssRNA but not that of (+)ssRNA. We found TRiC surrounding structures similar to DLPs. We also show TRiC subunits localize in VLSs composed of NSP5 with either NSP2 or VP2, where VP2 improves the recruitment of TRiC subunits into the VLS. The inhibition of TRiC resulted in defective VLS when induced by NSP5 and VP2 but in intact VLS when induced with NSP5 and NSP2. Consistently, VP2 and NSP5 are associated with TRiC subunits as denoted by immunoprecipitation experiments.

The biochemical features of a substrate recognized by TRiC are poorly understood. MRV σ3 and VP2 share some biochemical features that could hint at VP2 as a suitable substrate for TRiC. For example, the presence of conserved and complex beta-sheets in VP2 central and apical domains (69) suits the conditions already described for other beta-sheet-rich substrates like tubulin or actin (46). Alternatively, TRiC may stabilize higher-order structures of VP2, favoring primed forms and allowing later association with VP1 and formation of the core-shell particles. In this sense, TRiC can retain a polypeptide in an almost native state until it binds to a protein interactor or a co-chaperone, such as Hsp70, to assist in the folding of higher-order structures (70, 71).

The RdRp of RV, VP1, has a double task of transcription and replication. When transcribing, VP1 within DLPs uses (−)ssRNA as a template for synthesizing (+)ssRNA, which is required for the translation of RV proteins but also as a template for replication of its genome segments. It is thought that transcription occurs in two waves (72, 73). The first transcription wave occurs immediately after internalization when transcriptionally active DLPs are released in the cytosol, permitting the translation of RV proteins required for halting host innate immunity, building the viroplasms, and shutting off host translation. The second wave of transcription occurs in the viroplasms, where the generated transcripts are released to the cytosol for translation by ribosomes (72, 74). The replication, corresponding to the synthesis of dsRNA genome segments, occurs only in the viroplasms. Although the strict requirement of VP2 as a cofactor for VP1 to initiate the dsRNA synthesis has been demonstrated by *in vitro* experiments (6, 75), no evidence has been provided in RV-infected cells. Moreover, high-resolution cryo-electron microscopy of purified RV particles showed that the C-terminal plug of VP1 and N-terminus of VP2 coordinate the fine-tuning of transcription/replication activities (40, 41). The replication and packaging of each of the 11 genome segments in the new core shell is a fine-tuned not yet elucidated mechanism occurring in viroplasms.

The contribution of NSP5 to the replication and packaging of the genome segments also needs to be considered. In this study, NSP5 associates with TRiC and localizes in viroplasms surrounding DLP-like structures. Since NSP5 has been demonstrated to be associated directly with VP1 (59) and bind RNA (76), we cannot discard the possibility that NSP5 is involved in coordinating RV replication by mediating association with TRiC. In this sense, an NSP5/KO recombinant RV is totally unable to replicate (7). Similarly, other RV proteins may depend on TRiC for folding. In fact, upon TRiC inhibition, NSP3, which is not directly associated with viroplasms, is redistributed in the cytosol, forming small aggregates, an indicator for misfolded proteins.

The current model for RV virion assembly (72) proposes a polymerase complex of VP1 and VP3 (PC) that associates in the viroplasms, in which VP1 interacts with the 3′ consensus sequence of each of the 11 (+) ssRNAs (42, 77). In parallel, VP2 self-assembles forming decamers, allowing the concomitant formation of core shells and the recruitment of the PC complex, enabling VP1 replication activity (6). Our results are consistent with the currently proposed model for RV assembly and genome packaging (78). Interestingly, we found DLP/TLPs empty of dsRNA genome and PC upon TRiC inhibition. In this situation, we observe that VP2 can still form core-shell particles. This result can be explained by TRiC coordinating the association of VP2 with PC. It is well known that VP2 forms spontaneous core-shell structures (79, 80). TRiC, therefore, would impede the immediate formation of VP2 core-shell particles by holding its higher-order structure (70, 71), as VP2 decamers, allowing the association of PC with the VP2 N-terminus localized in proximity to the fivefold axes (81). This process can eventually be assisted by other chaperonins like Hsp70 (82). Thus, the inactive TRiC may be unable to hold the VP2 oligomers and prevent the formation of spontaneous empty VP2 core shells. Also, we cannot discard the possibility that VP2 folding depends partially on TRiC. The VP2 core-shell domain seems independent of TRiC since it forms even upon inhibition of TRiC. However, the ability to associate with PC could depend on TRiC, as denoted by the lack of this complex in the Cryo-EM analysis of the TRICi-purified particles. A third option is the TRiC folding requirements for VP1. An unfolded VP1 would have impaired association with VP2 and replication activity. Finally, TRiC could be involved in folding an unknown host component, assisting in the assembly/packaging of the core shell. All these possibilities permit the association of the (+)ssRNAs with the PC by NSP5 (76) and NSP2 (8). NSP5 can then associate with VP1 (59) and TRiC (this study). VP6 is incorporated in the second layer to form the DLPs that bud to the ER to be converted into mature TLPs (83). As denoted by our results, the core-shell structure is preserved upon inhibition of TRiC and does not disturb the association of VP6, hence generating empty DLPs and TLPs.

Our results reinforce the role of VP2 in viroplasm formation (18, 19) since it is required to provide a globular morphology as denoted by irregularly shaped viroplasms and VLSs upon TRICi treatment. We cannot discard that distinct pools of VP2 in the viroplasms are dedicated to core-shell formation and building of viroplasms. Also, our direct RNA sequencing results indicate that most of the observed (+) ssRNAs must be provided by incoming DLPs, which is consistent with the collected time of the RNA samples (6 hpi), a time at which secondary transcription is still not robust (74). However, we cannot discard that a reduced and indistinguishable fraction is produced in the viroplasm. We are currently studying this possibility in our laboratory.

This study provides evidence of the role of TRiC/CCT in coordinating the assembly and packaging of the dsRNA genome segments and PCs in core-shell particles in the RV viroplasms.

## MATERIALS AND METHODS

### Cells and viruses

MA104 cells (embryonic rhesus monkey kidney, ATCCCRL-2378, RRID: CVCL_3845) were cultured in Dulbecco’s modified Eagle’s media (DMEM, GibcoBRL) supplemented with 10% fetal calf serum (FCS) (AMIMED, BioConcept, Switzerland) and penicillin (100 U/mL)-streptomycin (100 µg/mL)(Gibco, Life Technologies).

The MA104/NSP5-BioID2 cell line was generated using a lentiviral system. Briefly, HEK293T cells were maintained in DMEM (Life Technologies) supplemented with 10% FBS (Life Technologies) and 50 µg/mL gentamycin (Biochrom AG). Approximately 7 × 10^6^ HEK293T cells (human embryonic kidney, RRID: CVCL_0063) were seeded in a 10 cm^2^ tissue culture dish 24 h before transfection. For each well, 2.4 µg of pMD2-VSV-G, 4 µg of pMDLg pRRE, 1.8 µg of pRSV-Rev, and 1.5 µg of pAIP-NSP5-BioID2 were co-transfected with Lipofectamine 3000 (Sigma-Aldrich) according to the manufacturer’s instructions. After 48 h, the virus was collected and filtered with a 0.45 µm polyvinylidene fluoride filter. The virus stock was immediately used or stored at −80°C. For lentiviral transduction, MA104 cells were transduced in 6-well plates with 1 mL of lentiviral supernatant for 2 days. The positive cells were selected in 2 µg/mL puromycin. BHK-T_7/9_ (baby hamster kidney stably expressing T_7_ RNA polymerase) cells were kindly provided by Naoto Ito (Gifu University, Japan) (84) and cultured in Glasgow medium supplemented with 5% FCS, 10% tryptose phosphate broth (Sigma-Aldrich), 10% FCS, penicillin (100 U/mL)-streptomycin (100  µg/mL), 2% nonessential amino acids, and 1% glutamine.

Rotavirus porcine OSU strain (G5; P[9]), simian SA11 strain (G3; P6[1]), and rhesus RRV strain (G3; P5B[3]) were propagated in MA104 cells, as described previously (85). Virus titer was determined as described previously by Eichwald et al. (21) and expressed as viroplasm-forming units (VFU) per milliliter.

### Antibodies and reagents

Guinea pig anti-NSP5, guinea pig anti-NSP2, mouse anti-NSP2, guinea pig anti-VP2, guinea pig anti-VP1, and mouse scFV anti-NSP5 clone 1F2 were described previously (15, 16, 59, 86). Mouse monoclonal (mAb) anti-VP6 (clone 2F) was a gift from Dr. N. Mattion (CEVAN, Buenos Aires, Argentina). Mouse mAb anti-VP7 (clone 159), mouse anti-VP5 (clone 4G2), and mouse mAb anti-VP2 clone (3E8) were kindly provided by Harry B. Greenberg (Stanford University, CA, USA). Rabbit anti-NSP3 and mouse anti-VP1 were kindly provided by Susana López (UNAM, Mexico). Rabbit anti-NSP4 was kindly provided by Daniel Luque (ISCIII, Madrid, Spain). Rabbit anti-CCT1 was purchased at Invitrogen. Rabbit anti-CCT2 and rabbit polyclonal anti-CCT3 (A6547) were purchased at Abclonal. Goat anti-mouse conjugated to 6 nm colloidal gold particles and goat anti-rabbit conjugated to 12 nm colloidal gold particles were purchased from Jackson ImmunoResearch Europe Ltd. Mouse anti-GAPDH (clone GAPDH-71.1, RRID: AB_1078991) was purchased from Sigma-Aldrich. Mouse monoclonal anti-V5 was purchased at Abcam. Rat anti-Histone H3 phosphorylated Ser 328 (clone HTA28) S28P-Alexa 647 (RRID: AB_397065) was purchased at Biolegend. Rabbit anti-cdc20 (RRID: AB_890558) was purchased at Bethyl Laboratories, Inc. Mouse monoclonal anti-dsRed2 (RRID: AB_1562589) was purchased at Santa Cruz Biotechnology, Inc. Streptavidin-Dylight 488 was purchased at Invitrogen. Streptavidin-HRP was purchased at Sigma-Aldrich.

Paclitaxel (taxol) was purchased from Sigma-Aldrich. UBEI-41 was purchased from Nova Biologicals. TRICi corresponds to 2-[(4- chloro-2λ4,1,3-benzothiadiazol-5-yl)oxy]acetic acid (STK526585), which was chemically synthesized at Vitas M Chemical (62).

### DNA plasmids

pMD2.G (Addgene plasmid #12259, RRID: Addgene_12259), pMDLg/pRRE (Addgene plasmid# 12251, RRID: Addgene_12251), and pRSV-Rev (Addgene plasmid#12253, RRID: Addgene_12253) were a gift from Didier Trono (87). pAIP (Addgene plasmid #74171, RRID:Addgene_74171) was a gift from Jeremy Luban (88).

pAIP-NSP5-BioID2 was prepared by ligation of NSP5-BioID2 fragment in pAIP within NotI and EcoRI restriction enzymes. NSP5-BiolD2 was synthesized as a gene block by Genscript. pcDNA-V5-VP1, pcDNA-VP1, pcDNA-VP2, pcDNA-NSP5, pcDNA-NSP3, pcDNA-VP4, pcDNA-VP7, and pcDNA-VP6 were previously described (16, 18, 59, 89).

### Pull-down assay

MA-NSP5-BioID2 cells (5 × 10^6^) were infected with RV-SA11 (MOI of 5 VFU/cell). For biotin labeling, cells were immediately washed after adsorption with phosphate-buffered saline (PBS). Next, the media were replaced by DMEM supplemented with 10% FBS, 50 µg/mL gentamycin, and 200 µM biotin (Merck) and incubated for 17 h at 37°C. Subsequently, the cells were gently washed once in PBS and then lysed in lysis buffer (50 mM Tris pH 8.0, 500 mM NaCl, and 0.1 mM EDTA). The cell lysate was harvested in a 1.5 mL tube and centrifuged at 13,000 rpm for 1 min at 4°C. Next, the supernatant was collected and incubated with 40 µL of Streptavidin Mag Sepharose (GE Healthcare Life Sciences) in the rotator wheel for 1 h at 4°C. The beads were subsequently washed three times with 500 µL of lysis buffer supplemented with 0.5% SDS, three times with lysis buffer supplemented with 1% NP-40, and three times with lysis buffer. The beads were then recovered and used for the downstream experiments.

For reverse pull-down assay, 2.4 × 10^6^ MA104/NSP5-BioID2 cells were RV infected at an MOI of 25 VFU/cell. At 1 hpi, media were replaced by media containing 100 µM biotin in serum-free DMEM. The cells were harvested at 6 hpi by detaching the cells with 5 mM EGTA in PBS and spun down for 2 min at 1,500 rpm. The cellular pellet was resuspended in 2.5 mL of ice-cold ATP-depletion buffer (1 mM sodium azide, 2 mM 2-deoxyglucose, 5 mM EDTA, and 5 mM cycloheximide in PBS without calcium and magnesium) and then centrifuged at 300 × *g* for 5 min at 4°C. The cell pellet was resuspended in 1 mL lysis buffer B (50 mM HEPES-KOH pH 7.5, 100 mM KCl, 5 mM EDTA, and 10% glycerol) supplemented with a protease inhibitor cocktail (Roche, Switzerland). Next, the cells were lysed by freezing and thawing three times, followed by Dounce homogenization (70 strokes). The cell lysates were clarified by centrifugation at 17,000 × *g* for 10 min at 4°C. The cell lysate was dialyzed overnight at 4°C against PBS using a Slide-A-Lyser mini dialysis cassette (MWCO 3500, Thermo Fisher Scientific). For the input, 50 µL cell lysate was collected. The cell lysate was mixed with 1 volume of lysis buffer B supplemented with 2% BSA and 50 µL of Dynabeads M-270 Streptavidin (Thermo Fisher Scientific, cat 65305) and incubated at 4°C for 30 min with rotation. The beads were equilibrated in lysis buffer B supplemented with 2% BSA and incubated in wheel for 30 min at 4°C. The bead-bound complexes were washed three times with 500 µL of ice-cold TRiC wash buffer (50 mM HEPES-KOH pH 7.5, 100 mM KCl, 5 mM EDTA, 10% glycerol, and 0.05% NP-40), eluted with 20 µL SDS sample buffer, and resolved by SDS-PAGE.

### Mass spectrometry and proteomics

MA104/NSP5-BioID2 cells (1.5 × 10^8^) were infected with SA11 (MOI, 5), treated at 6 hpi with 100 µM biotin, and lysed 24 h post-transfection in TNN buffer [Tris–HCl 100  mM (pH 8), NaCl 250  mM, and NP-40 0.5% with cOmplete protease inhibitor cocktail (Roche)]. The pull-down of biotinylated proteins was performed using 100 µL StrAv Mag Sepharose (GE Healthcare) for each condition and incubated in a rotation wheel for 3 h at 4°C. For mass spectrometry analysis, the washed biotin pull-downs were digested directly with trypsin (200 ng) in 20 µL of 20 mM triethyl ammonium bicarbonate pH 8.5 for 16 h at room temperature. The supernatant was removed, the beads were washed once with 50 µL of 0.1% formic acid, and the two fractions were pooled and concentrated using STAGE tips (90). The samples were resuspended in 10 µL of 0.1% formic acid and analyzed by LC-MS/MS using a NanoEASY LC (Thermo) coupled with an amaZon ETD ion trap (Bruker Daltonics). The resulting spectra were searched against the human and rotavirus proteomes using the GPM (91). Results were filtered to remove all results with an *e*-value > 0.05. Statistical analysis and plots were performed using R (4.1.3). The figure was finalized in Adobe Illustrator 2022.

### Immunofluorescence

Samples were processed for immunofluorescence as described in detail by Vetter et al. (29). Images were acquired at CLSM and processed with imageJ2 version 2.3.0/1.53q (Creative Commons license).

For native detection of antigens in RV TLPs, coverslips were pre-embedded with 50 µg/mL fibronectin in PBS, incubated for 30 min at room temperature (92), and rinsed once with PBS. The purified TLPs, corresponding to 2.5 × 10^6^ VFU, were diluted in 200 µL of TBS (137 mM NaCl, 5 mM KCl, 7 mM Na_2_HPO_4_, 5.55 mM dextrose, 25 mM Tris pH 7.4, 1 mM MgCl_2_, and 1 mM CaCl_2_) and incubated for 60 min at room temperature. The samples were fixed with 2% paraformaldehyde in PBS for 10 min at room temperature, washed three times with 0.1% triton X-100 in PBS, and blocked with 1% BSA in PBS for 20 min at room temperature. The coverslips were incubated with primary antibody diluted 1:100 and secondary antibody diluted 1:500. Samples were mounted in Prolong Gold (Thermo Fisher Scientific) and examined at LSCM Leica SP8 inverse.

### dsRNA genome segments’ extraction and electropherotype

MA104 cells seeded at a density of 5 × 10^5^ in a 60 mm tissue culture plate were reverse transfected with 10 nM of indicated siRNA and 10 µL of Lipofectamine RNAiMAX Transfection Reagent (ThermoFisher Scientific) in a final volume of 5 mL as described above. At 48 hpt, cells were RV-infected (MOI, 25 VFU/cell). For this purpose, cell monolayers were washed once with phosphate-buffered saline (137 mM NaCl, 2.7 mM KCl, 8 mM Na_2_HPO_4_, and 2 mM KH_2_PO_4_ pH 7.4) and adsorbed with 500 µL of diluted virus for 1 h at 4°C. Then, 3 mL of serum-free DMEM was added to the cells and incubated at 37°C. At 6 hpi, media were removed, and cells were harvested in 500 µL of RNA extraction buffer (0.5% NP-40, 150 mM NaCl, 1.5 mM MgCl_2_, and 10 mM Tris pH 7.4) in a 1.5 mL tube.

Then, the dsRNA genome was extracted with 500 µL of phenol-chloroform pH 4.6, followed by ethanol-sodium acetate precipitation (93). The pellet was resuspended in 20 µL of distilled water and mixed with 18 µL gel loading dye (New England Biolabs, Inc.). Samples were resolved in a 10% SDS-polyacrylamide gel, and dsRNA genome segments were stained with GelRed (Biotium) and visualized at Odyssey Fc Imager (LI-COR Biosciences).

We implemented a highly sensitive method based on genomic DNA TapeStation (Fig. S7a through d) (sensitivity range of 0.5 ng/µL) to determine the presence of RV dsRNA genome extracted from the purified subviral particles. For this purpose, precipitated dsRNA genome segments of CsCl purified particles were resuspended in 15 µL of nuclease-free water. Then, 2 µL of the sample was mixed with 10 µL of sample buffer for genomic DNA (5067-5366, Agilent), loaded on genomic DNA ScreenTape with a size range from 200 to >60,000 bp (5067-5365, Agilent), and migrated in the Agilent TapeStation 4150 (Agilent). The data were analyzed using TapeStation Analysis software 3.2 (Agilent).

### Immune and transmission electron microscopy

For immune electron microscopy, three confluent T-75 flasks of MA104 cells per sample were OSU-infected (MOI, 50 VFU/cell). For this purpose, the virus was adsorbed for 1 h at 4°C and incubated in serum-free DMEM for 5.5 hpi at 37°C. Then, the cells were incubated for 30 min with media containing 10 µM Taxol. At 6 hpi, the cells were released with trypsin, harvested in a complete medium, and spun down at 1,500 rpm for 2 min at room temperature. The cellular pellets were prepared according to the Tokuyasu method (94). Briefly, the cells were fixed with 4% formaldehyde at room temperature, washed several times with 0.1 M Na-phosphate, pH 7.4, and pelleted for 3 min at 13,000 rpm and 37°C in 12% gelatin. The gelatin-embedded blocks immersed in 2.3 M sucrose were kept overnight at 4°C, mounted on ultramicrotome specimen holders (UC6, Leica Microsystems, Wetzlar, Germany), and frozen by plunging into liquid nitrogen. After trimming to a suitable block size and shape, 70 nm sections were cut at −120°C using a dry diamond knife (Diatome, Biel, Switzerland). Flat ribbons were picked up with a wire loop filled with a drop composed of 1% methylcellulose, 1.15 M sucrose in 0.1 M Na-phosphate, pH 7.4, and transferred onto carbon-coated formvar films mounted on 100 hexagonal mesh/inch copper grids. For antigen retrieval, the sections were incubated for 1 h at 40°C with 0.1 Na-phosphate buffer pH 5.5, washed with 50 mM glycine, blocked with 1% BSA, and incubated with mouse monoclonal anti-NSP5 (clone 1F2) or mouse monoclonal anti-VP6 (clone 2F) at a dilution of 1:1 and rabbit anti-CCT3 (Abclonal), rabbit polyclonal anti-CCT2 (Abclonal), or rabbit monoclonal anti-CCT2 (Abcam) at a dilution of 1:5 at room temperatures for 90 min, washed several times with 0.1% BSA. Then, the samples were incubated for 45 min at room temperature with a goat anti-mouse antibody conjugated with 6 nm colloidal gold particles and a goat anti-rabbit antibody conjugated with 12 nm colloidal gold particles (Jackson ImmunoResearch Laboratories, Inc., West Grove, PA, USA). After incubation, the samples were washed with 0.1 M Na-phosphate, pH 7.4, and distilled water and transferred to a mixture of 1.8% methylcellulose and 0.4% uranyl acetate. After 5-min incubation, the grids were looped out, and the excess solution was drained and air-dried to obtain a thin film on the grid.

For transmission electron microscopy images, MA104 cells were seeded at a density of 2 × 10^4^ cells per well in a 24-well tissue culture plate, and the sapphire discs were then immediately added. At 48-h post-seeding, the cells were RV-infected at an MOI of 75 VFU/cell and at 1 hpi untreated or treated 1.25 or 2.5 mM TRICi. The cells were fixed at 6 hpi with 2.5% glutaraldehyde in 100 mM Na/K phosphate buffer, pH 7.4, for 1 h at 4°C and kept in 100 mM Na/K phosphate buffer overnight at 4°C. Afterward, the samples were post-fixed in 1% osmium tetroxide in 100 mM Na/K phosphate buffer for 1 h at 4°C and dehydrated in a graded ethanol series starting at 70%, followed by two changes in acetone, and embedded in Epon. Ultrathin sections (60–80 nm) were cut and stained with uranyl acetate and lead citrate.

For negative staining, the RV particles were adsorbed for 10 min on glow-discharged carbon-coated Parlodion films mounted on 300 mesh per inch copper grids (Electron Microscopy Science, Hatfield, PA, USA). Samples were washed once with distilled water and stained with saturated uranyl acetate (Fluka) for 1 min at RT. For the calculation of the diameter of virus particles by negative staining, the area of each virus particle was calculated using Imaris software (version 2.1.0/1.53c; Creative Commons license) and then converted to the diameter as follows: $d=2\times \sqrt{(}A/\pi )$, where *A* is the area and *d* is the diameter of the particle, respectively.

All the samples were acquired in a transmission electron microscope (CM12; Philips, Eindhoven, The Netherlands) equipped with a charge-coupled-device camera (Orius SC1000A 1; Gatan, Pleasanton, CA, USA) run with a Digital Micrograph software (Gatan) at an acceleration of 100 kV. The images were processed for publication using Image J (version 2.0.0-rc-69/2.52p)

### Quantification of viroplasms and VLSs

Viroplasm size, number, and frequency were quantified previously (21, 28). The data were processed using Microsoft Excel for Mac (version 16.61.1). Statistical analysis, unpaired parametric two-way ANOVA, and plots were performed using Prism 10 for macOS version 10.0.0 (131) (GraphPad Software, LLC).

### Quantification of protein signal in VLS

The fluorescence signal values in the VLSs were determined similarly, as described previously by Eichwald et al. (95). The intensity profile of a linear region of interest was obtained using the ImageJ plot profile tool. The co-localization value was obtained by calculating the area below the curve of intensity profiles of both NSP5 VLS and other proteins. For this purpose, the Image J magic wand tool was used to provide the gray value intensity for each point, from which a protein signal percentage was obtained on the occupied NSP5-VLS signal area below the curve. The CCT3 normalized value from the area under the curve was obtained using the following formula:


Normalized CCT3 value=[(CCT3/NSP5)−(CCT3/NSP5)min]/[(CCT3/NSP5)max−(CCT3/NSP5)min]

where CCT3 and NSP5 correspond to the value obtained from the area under the curve for each VLS. The minimal (min) and maximal (max) signal values for each condition were obtained from all the experimental tested samples.

### Immunoblotting

Cells seeded in 12 multiwell tissue culture plates at a density of 2 × 10^5^ cells per well were lysed directly by adding 25 µL of Laemmli sample buffer 4× (8% SDS, 40% glycerol, 200 mM Tris-HCl pH 6.8, and 0.4% bromophenol blue). The cell extracts were heated for 5 min at 95°C, sonicated for 5 s at 14 Hz, and loaded in SDS-polyacrylamide gel. The proteins were migrated at 30 mA per gel and successively transferred to 0.45 µm Protran nitrocellulose membrane (Amersham). The membranes were blocked for 30 min in 5% milk-PBS and then incubated with primary and the corresponding secondary antibody conjugated to IRdye 680 or ICW780 (LI-COR). Samples were acquired at Odyssey Fc Imager (LI-COR Biosciences).

### Purification of RV subviral particles by CsCl gradient

Fifty T-150 tissue culture flasks with confluent MA104 cells were used for each experimental point. The cells were washed once with PBS and then infected with RV porcine OSU strain at an MOI of 25 VFU/cell diluted in 5 mL of serum-free DMEM per flask. The virus was adsorbed for 1 h at 4°C with gentle mixing. After adding 15 mL of DMEM serum per flask, the cells were transferred to 37°C. At 1 hpi, the media were removed, and the cells were washed with 5 mL of serum-free DMEM per flask, followed by adding 10 mL of complete media supplemented with 2.5 mM TRICi. At 8 hpi, the cells were washed with 5 mL PBS and released with 1 mL trypsin-EDTA 0.5% per flask. The cells were harvested in complete DMEM and centrifuged for 5 min at 1,500 rpm and 4°C. Then, the cells were washed once more with PBS and stored at −80°C. The cellular pellets were resuspended in 15 mL of TNC (10 mM Tris-HCl, 140 mM NaCl, and 10 mM CaCl_2_) with Pierce protease inhibitor (Thermo Scientific), Dounce homogenized with 20 strokes, sonicated three times for 10 s at 20% amplitude, and centrifuged for 10 min at 12,500 *× g* and 4°C. The supernatant was recovered and loaded over a 10 mL of 25% sucrose cushion in TNC buffer using SW 28Ti rotor (Beckman Coulter) and centrifuged for 2 h at 100,000 × *g* and 4°C. The pellet was resuspended overnight at 4°C with 2.5 mL of TNC buffer plus protease inhibitor. Then, the samples were diluted to a final volume of 5 mL and extracted with 5 mL of Vertrel XF (1,1,1,2,2,3,4,5,5,5-decafluoropentane, Dupont). The aqueous phase was diluted to 14 mL in a final 1.37 g/mL CsCl solution. The samples were centrifuged in NVT 65 rotor (Beckman Coulter) for 24 h at 45,000 *× g* at 4°C. The indicated fractions were collected and dialyzed against TNC buffer overnight at 4°C. Then, the samples were spun down using a SW60 Ti rotor (Beckman Coulter) for 1 h at 250,000 *× g* and 4°C. The pellet was resuspended in 25 µL TNC buffer.

### Cryo-EM and data collection

Purified TRICi fraction 4 sample (5 µL) was applied onto R2/2 300 mesh Quantifoil Cu/Rh grids and vitrified using a ThermoFisher Scientific Vitrobot Mark IV automatic plunger. Data were collected on an FEI Talos Arctica electron microscope operated at 200 kV, and images were recorded on an FEI Falcon III detector. A total of 2,369 movies were recorded with a total exposure dose of 40.8 e^-^ Å^−2^ divided into 40 frames and a calibrated pixel size of 1.37 Å on the specimen. Data acquisition was performed with EPU Automated Data Acquisition Software for Single Particle Analysis (ThermoFisher Scientific) at −0.70 to −3.0 µm defocus.

### Cryo-EM image processing

All image-processing steps were performed inside CryoSPARC (96) and Scipion (97) package software. Movies were motion-corrected and dose-weighted with Motioncor2 (98). Aligned non-dose-weighted micrographs were then used to estimate the contrast transfer function (CTF) with the CryoSPARC patch CTF routine. A small data set of particles (200) was manually picked and used to generate 2D averages that were used as references to pick the averaged micrographs automatically, and a total of 3,270 particles were extracted. Iterative CryoSPARC 2D classification was performed to select 1,885 particles, and the CryoSPARC *ab initio* routine combined with heterogeneous refinement was used to obtain initial 3D models for TLP and DLP containing 553 and 1,267 particles, respectively. These 3D reconstructions were refined using the CryoSPARC homogeneous refinement routine to 6.9 and 6.7 Å based on the gold-standard (FSC = 0.143) criterion. Images were generated using UCSF Chimera (99).

### Nanopore sequencing and data analysis

For extract preparation, MA104 cells were seeded at a density of 4 × 10^5^ cells/well in six multiwell plates. The next day, the cells were washed twice with PBS and then infected with porcine strain OSU (MOI of 12.5 VFU/cell) diluted in 250 µL of serum-free DMEM. After 1 h of adsorption at 4°C, 750 µL of serum-free DMEM was added to the well, and the cells were transferred to an incubator at 37°C. For experiments, including TRICi inhibitor, media were replaced at 1 hpi by adding 1.5 mL of serum-free media containing either 2% DMSO or 2.5 mM TRICi. At 6 hpi, the cells were lysed, and RNA was purified using an RNeasy mini kit (QIAGEN, Switzerland). Specifically, the genomic DNA was digested in-column using DNAse I as described by the manufacturer. RNA was eluted with 30 µL of nuclease-free water by incubating for 1 min before spinning down. Then, the column was re-eluted using 30 µL elution buffer. The RNA concentration and integrity were determined using 1 µL in 4150 TapeStation System (Agilent). The rest of the sample was immediately frozen at −80°C. For denaturation of dsRNA, the total purified RNA was heated for 5 min at 95°C and then immediately placed on ice. Then, the RNA sample was prepared as described in direct RNA sequencing protocol from Oxford Nanopore Technologies Limited, starting with an input of 1,350 ng of RNA and a specific rRTA. For this purpose, specific primers binding the 5′- and 3′-UTR of each RV genome segment and to GAPDH were synthesized at Microsynth AG (Switzerland) and are described in Table S1. Oligo A was diluted to 2.8 µM TN buffer (10 mM Tris-HCl pH 7.5 and 50 mM NaCl). Next, each oligo B, corresponding to RV 5′- and 3′-UTR of each genome segment, was diluted in TN buffer to 0.116 µM and pooled to reach a final concentration of 2.8 µM of total oligo B. Then, the oligos were mixed 1:1 and annealed in a PCR machine (95°C for 2 min and then −1°C per 15 s to 25°C). This oligonucleotide was used as a replacement for RTA in the nanopore protocol. The samples were loaded on MinION Mk1B and started with the base calling in real-time using Guppy base calling software version (6.0.7+c7819bc) (Oxford Nanopore Technologies Limited). The reads were mapped with Minimap2 software (100) to the genomic sequences of rotavirus A strain/porcine/CH/2020/OSU/serotype 5 (GenBank accession nos. MT066200.1, MT066201.1, MT066202.1, MT066203.1, MT066204.1, MT066205.1, MT066206.1, MT066207.1, MT066208.1, MT066209.1, and MT066210.1) followed by RStudio software version (2022.02.0+443) (RStudio: Integrated Development for R. RStudio, PBC, Boston, MA, USA, URL: https://www.rstudio.com/).

### Co-immunoprecipitation of TRiC

The day before transfection, BHK-T7 cells were seeded at a density of 4 × 10^5^ cells/well in six multiwell tissue culture plates for single protein expression. The cells were transfected with 6 µg of pcDNA-VP2, pcDNA-NSP5, or pcDNA-V5-VP1 and 72 µL of Lipofectamine 2000 (Invitrogen) following the manufacturer’s instructions. The transfected cells were harvested at 24 hpt. The immunoprecipitation was adapted from the protocol described by Knowlton et al. (55). The cell monolayer was detached by adding 5 mM EGTA in PBS and incubated for 10 min at 37°C. The cells were harvested in a 15 mL conical tube and spun down for 5 min at 1,500 rpm. The pellet was resuspended in 2.5 mL of ice-cold ATP-depletion buffer (1 mM sodium azide, 2 mM 2-deoxyglucose, 5 mM EDTA, 5 mM cyclohexamide in PBS without Ca^2+^ and Mg^2+^). Then, the cells were centrifuged at 300 × *g* for 5 min at 4°C. The cell pellet was resuspended in 1 mL of lysis buffer B (50 mM HEPES-KOH pH 7.5, 100 mM KCl, 5 mM EDTA, and 10% glycerol) supplemented with a protease inhibitor cocktail. The cells were lysed by freezing and thawing three times using liquid nitrogen and a 37°C water bath followed by Dounce homogenization (70 strokes). The cell lysate was clarified by centrifugation at 17,000 × *g* at 4°C for 10 min and transferred to a new 1.5 mL tube. The input corresponded to 50 µL of cell lysate. For immunoprecipitation, the cell lysate was split into two equal volumes and combined with 2 µg of rabbit CCT2 specific monoclonal antibody (Abcam, ab92746), 2 µg of rabbit anti-CCT3 (Ray Biotech, 144-06547-200), or 2 µg of IgG isotype control antibody (Abcam, ab172730) and incubated at 4°C for 30 min with rotation. The cell lysates were then combined with 50 µL of Protein G Dynabeads (ThermoFisher, 10004D) equilibrated lysate buffer B and re-incubated at 4°C for 30 min with rotation. The bead-bound antibody-antigen complexes were washed four times with 500 µL of ice-cold TRiC wash buffer (50 mM HEPES-KOH pH 7.5, 100 mM KCl, 5 mM EDTA, 10% glycerol, and 0.05% NP-40), eluted with SDS sample buffer, and resolved by SDS-PAGE. The proteins were detected by immunoblotting, as described above.

### Statistical analysis

Statistical analyses were performed using Prism 9 (version 9.4.1, GraphPad Software) and RStudio software (version 2022.02.0 + 443, PBC, Boston, MA, USA). The error bars and statistical tests are indicated for each corresponding experiment. *P* values > 0.05 were considered statistically significant (*P < 0.05; **P < 0.01; and ***P < 0.001).

## Data Availability

The RNA sequence coverage from ONT can be found in the SRA repository with the following accession reference: PRJNA900220. The lead contact will share all data reported in this publication upon request.

## SUPPLEMENTAL MATERIAL

Figure S1 mbio.00499-24-s0001.tiff TRiC antibodies do not crossreact with RV antigens.

Figure S2 mbio.00499-24-s0002.tiff Distribution of CCT1, CCT2, and CCT3 in MA104 cells and the TRiC subunit CCT2 colocalizes in viroplasms surrounding DLPs.

Figure S3 mbio.00499-24-s0003.tiff Characterization of TRICi in MA104 cells.

Figure S4 mbio.00499-24-s0004.tiff TRIC inhibition hampers viroplasm formation of RV strains OSU, SA11, and RRV and silencing CCT3 and CCT2 TRiC subunits decreases virus progeny.

Figure S5 mbio.00499-24-s0005.tiff TRICi effect is reversible over viroplasm formation.

Figure S6 mbio.00499-24-s0006.tiff *In vivo* NSP5 hyperphosphorylation assay in presence of TRICi and localization of TRiC components in VLSs.

Figure S7 mbio.00499-24-s0007.tiff Analysis of OSU subviral particles isolated from infected cell extract untreated or treated with 2.5 mM TRICi.

Supplemental information mbio.00499-24-s0008.docx Legends for Figures S1 to S7 and Tables S1 and S2; supplemental materials and methods, supplemental references.

Table S1 mbio.00499-24-s0009.docx Reverse transcriptase adaptors used for Oxford Nanopore Technology direct RNA sequencing of RV positive and negative single-stranded RNAs.

Table S2 mbio.00499-24-s0010.docx Summary of LC-MS/MS of bands extracted from CBS lanes of fraction 1 (DMSO) and fraction 4 (TRICi) of CsCl gradient and Halotag-HA-VP2 extracts migrated in native gel.

ASM does not own the copyrights to Supplemental Material that may be linked to, or accessed through, an article. The authors have granted ASM a non-exclusive, world-wide license to publish the Supplemental Material files. Please contact the corresponding author directly for reuse.
