# Supplementary figures and images for: The recruitment of TRiC chaperonin in rotavirus viroplasms correlates with virus replication
Source: mBio. 2024 Mar 12;15(4):e00499-24. doi: 10.1128/mbio.00499-24 (PMC11005421; doi:10.1128/mbio.00499-24)

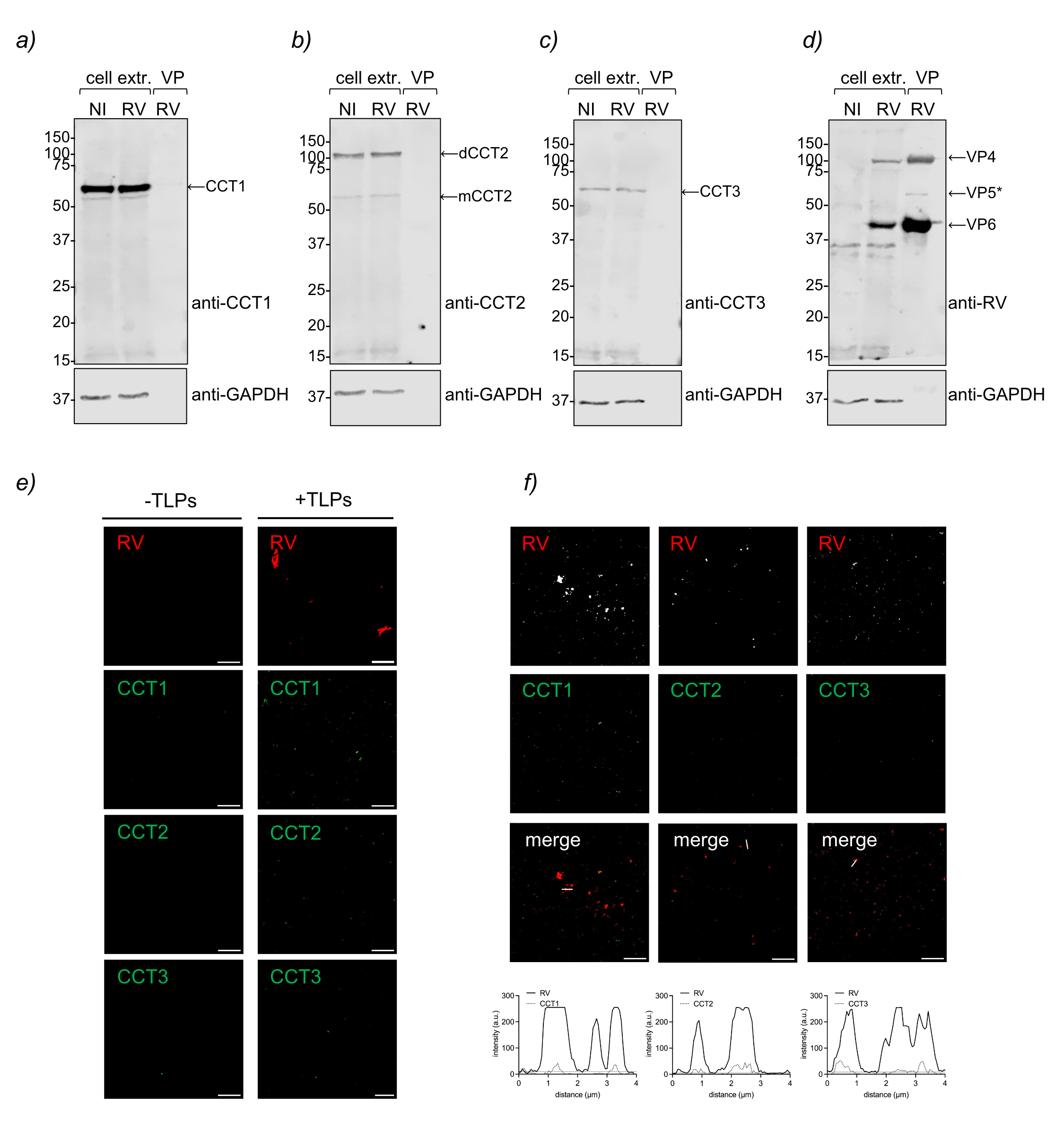

Supplement: Figure S1 — TRiC antibodies do not crossreact with RV antigens. [file mbio.00499-24-s0001.tiff]

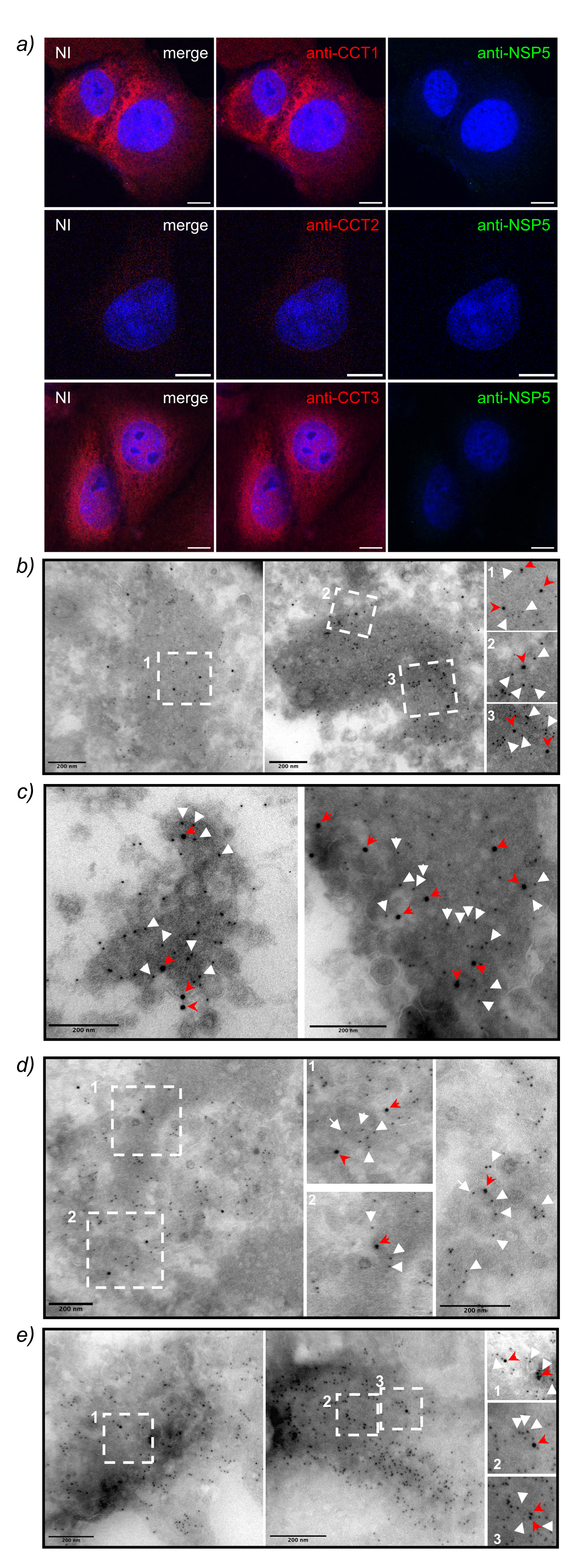

Supplement: Figure S2 — Distribution of CCT1, CCT2, and CCT3 in MA104 cells and the TRiC subunit CCT2 colocalizes in viroplasms surrounding DLPs. [file mbio.00499-24-s0002.tiff]

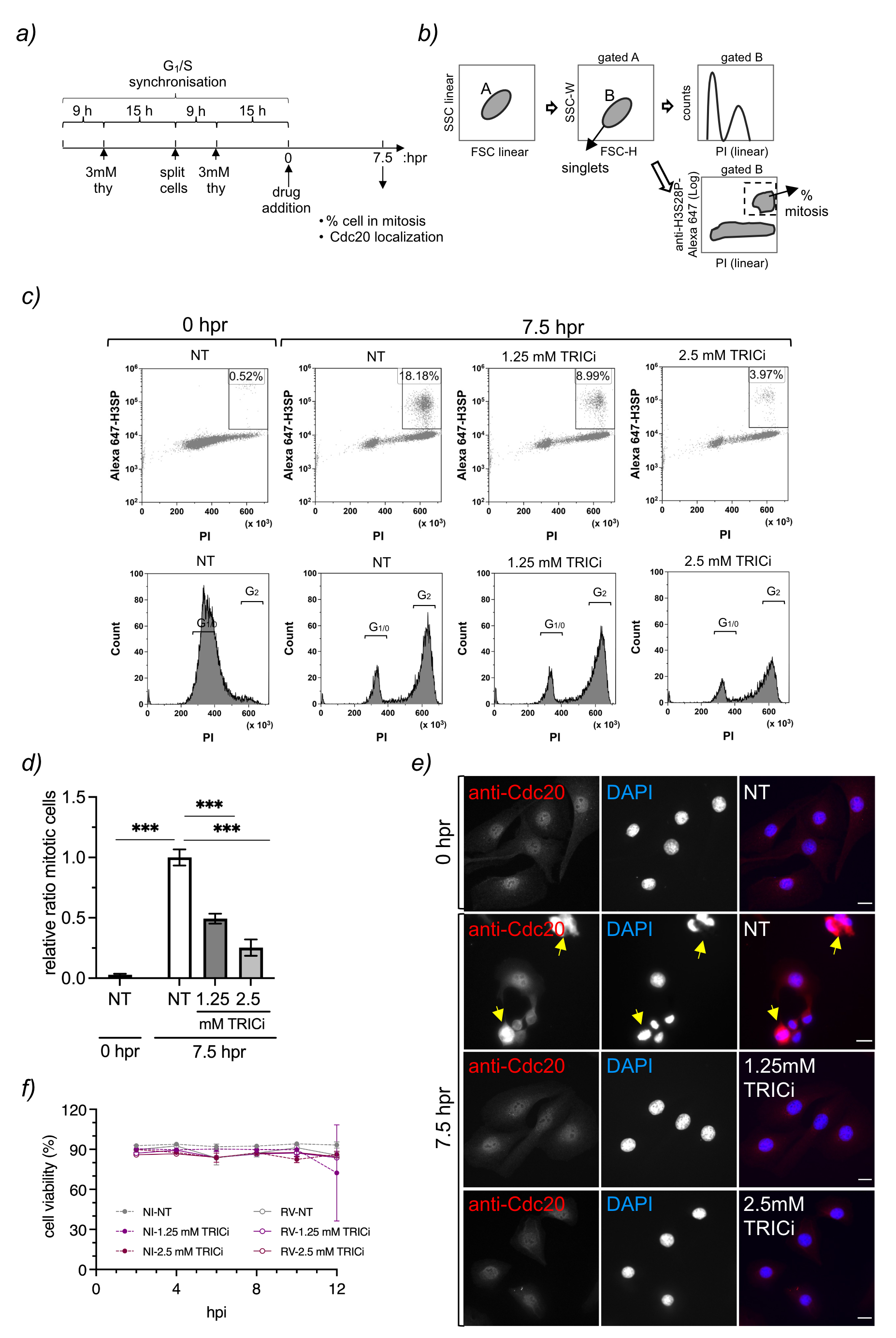

Supplement: Figure S3 — Characterization of TRICi in MA104 cells. [file mbio.00499-24-s0003.tiff]

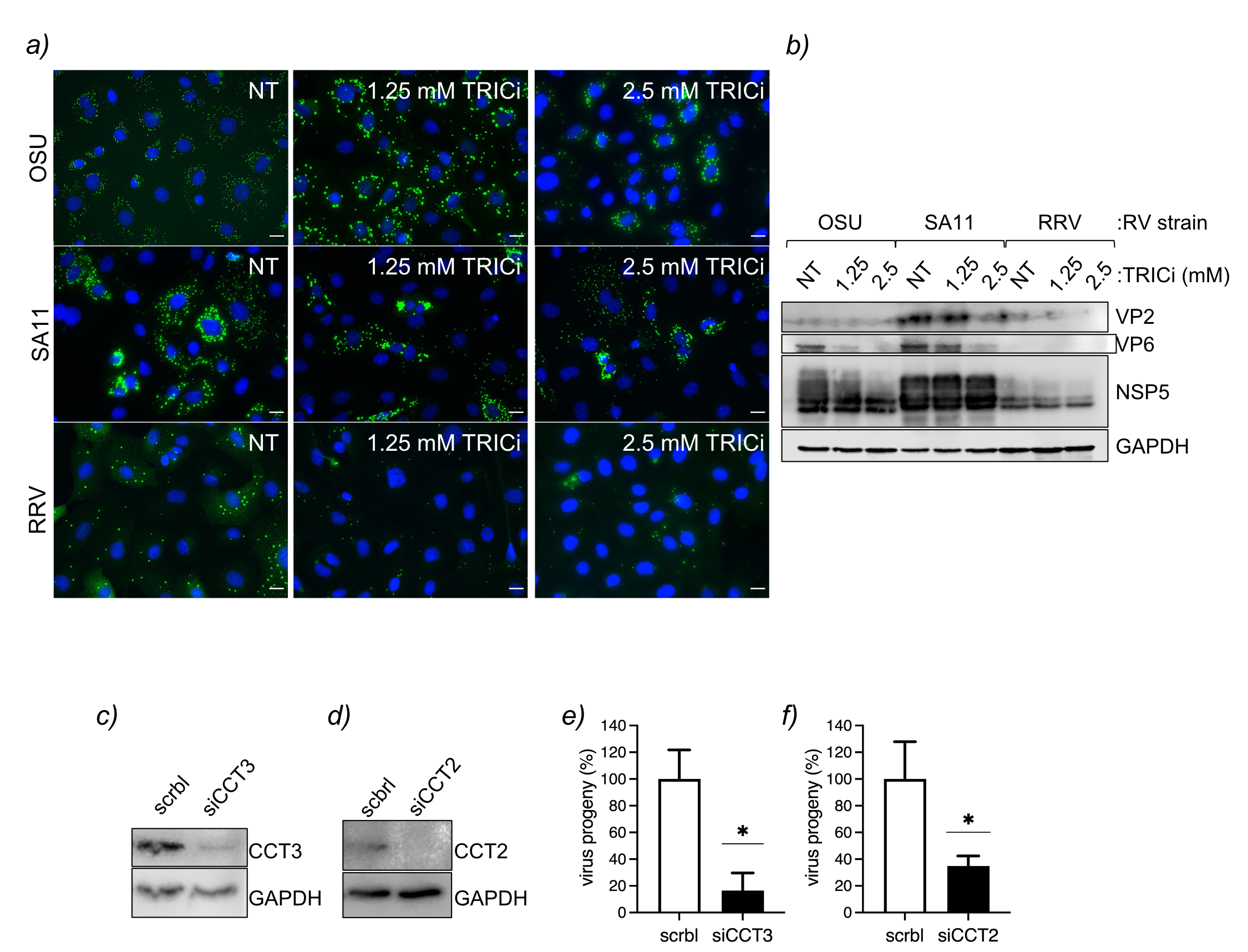

Supplement: Figure S4 — TRIC inhibition hampers viroplasm formation of RV strains OSU, SA11, and RRV and silencing CCT3 and CCT2 TRiC subunits decreases virus progeny. [file mbio.00499-24-s0004.tiff]

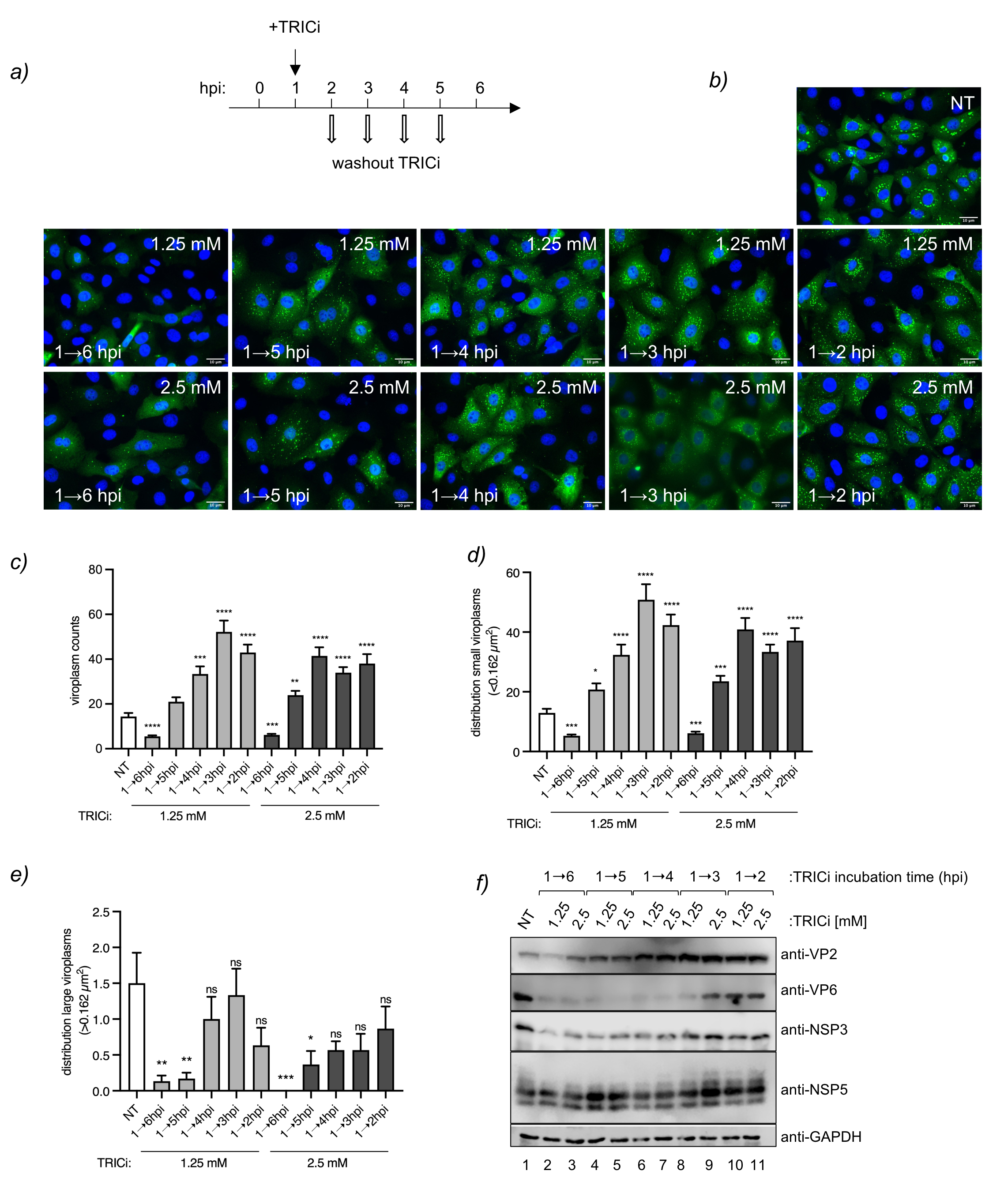

Supplement: Figure S5 — TRICi effect is reversible over viroplasm formation. [file mbio.00499-24-s0005.tiff]

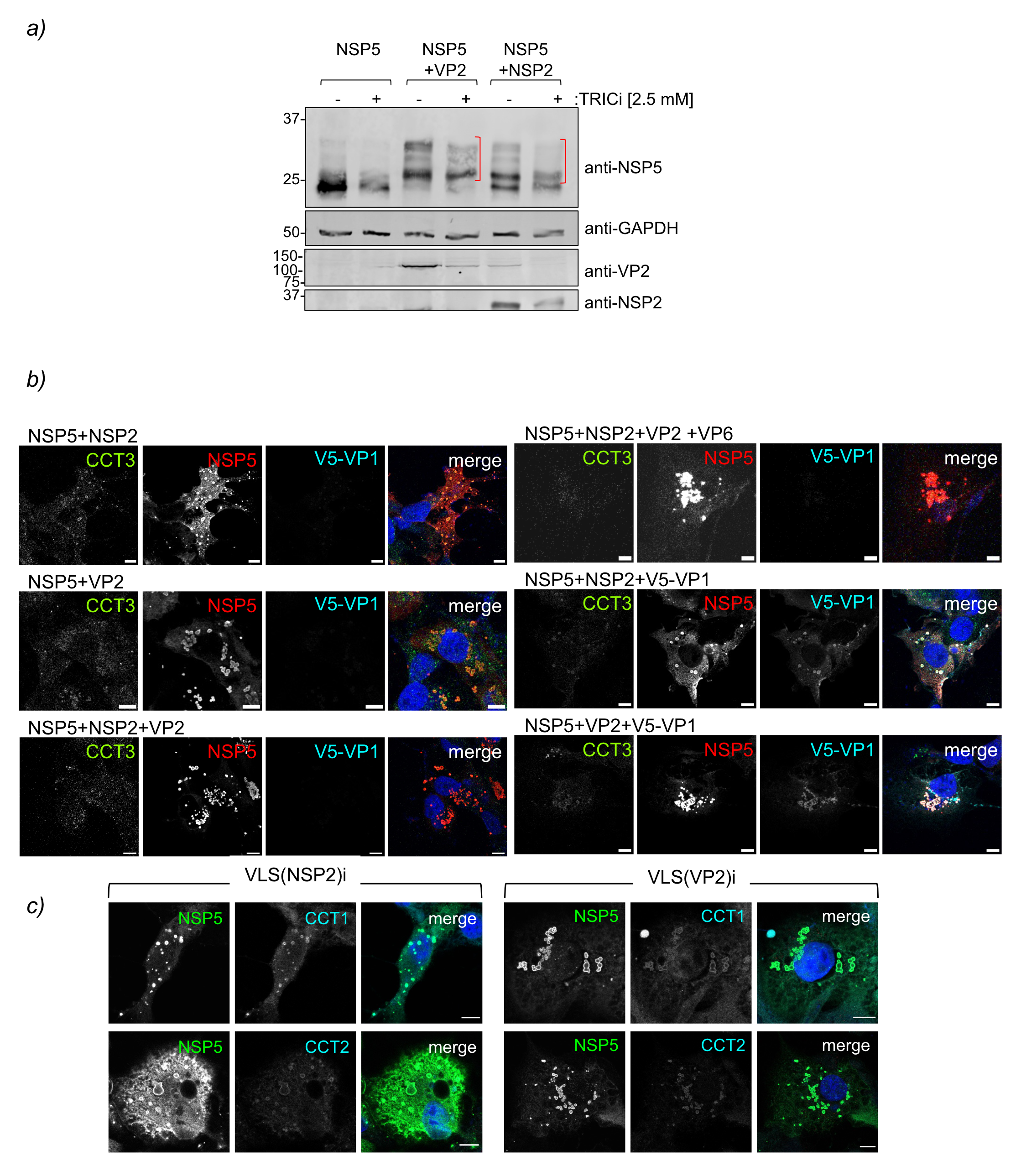

Supplement: Figure S6 — In vivo NSP5 hyperphosphorylation assay in presence of TRICi and localization of TRiC components in VLSs. [file mbio.00499-24-s0006.tiff]

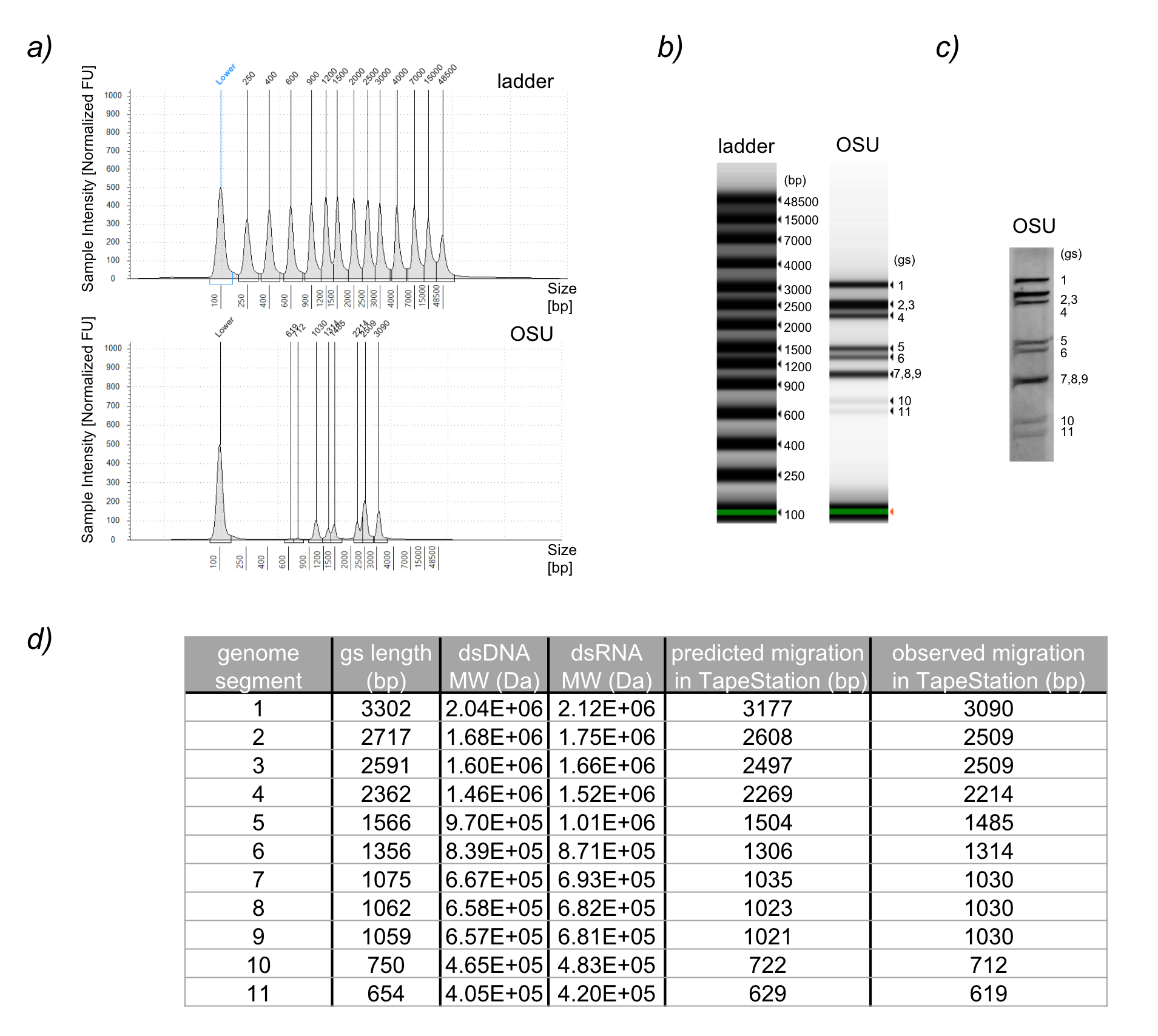

Supplement: Figure S7 — Analysis of OSU subviral particles isolated from infected cell extract untreated or treated with 2.5 mM TRICi. [file mbio.00499-24-s0007.tiff]
