# Supplementary material for: The recruitment of TRiC chaperonin in rotavirus viroplasms correlates with virus replication
Source: mBio. 2024 Mar 12;15(4):e00499-24. doi: 10.1128/mbio.00499-24 (PMC11005421; doi:10.1128/mbio.00499-24)
Supplement: Supplemental information — Legends for Figures S1 to S7 and Tables S1 and S2; supplemental materials and methods, supplemental references. [file mbio.00499-24-s0008.docx]

**Supplementary Information**

**Supplementary Figure Legends**

**Figure S1. TRiC antibodies do not crossreact with RV antigens**. Immunoblot membranes of non-infected (NI) and RV-infected MA104 cell extracts and RV virus particles (VP) incubated with rabbit polyclonal anti-CCT1 **(a)**, rabbit polyclonal anti-CCT2 **(b)**, rabbit polyclonal anti-CCT3 **(c)** and guinea pig anti-RV **(d)** antibodies. The monomer and dimer of CCT2 are pointed with arrows. The RV proteins are indicated. GAPDH is used as a loading control. **e)** Immunofluorescence images of slides pre-embedded with fibronectin and coated without and with TLPs (2.5x10^6^ VFU) followed by immunostaining with guinea pig anti-RV (red), rabbit anti-CCT1 (green), rabbit anti-CCT2 (green) or rabbit anti-CCT3 (green). The scale bar is 10 µm. **f)** Immunofluorescence images of TLPs coated on slides pre-embedded with fibronectin followed by co-immunostaining with guinea pig anti-RV (red) and rabbit anti-CCT1, CCT2 or CCT3(green). A merged picture is shown in the right column. The scale bar is 10 µm. The plots correspond to the intensity profile of the linear region of interest (LROI) indicated in the bottom image frame.

**Figure S2. Distribution of CCT1, CCT2, and CCT3 in MA104 cells and the TRiC subunit CCT2 colocalizes in viroplasms surrounding DLPs. a)** Immunofluorescence images showing the distribution of TRiC subunits CCT1, CCT2, and CCT3 in non-infected MA104 cells. After fixation with methanol, the cells were co-immunostained with indicated specific antibodies for detecting TRiC subunits (Alexa 594, red) and anti-NSP5 (Alexa 488, green). Nuclei were stained with DAPI (blue). The scale bar is 10 µm. Immune-electron microscopy of viroplasm fixed at 6 hpi. The thin sections were co-immunostained with either anti-NSP5 conjugated to 6 nm gold **(b and c)** or anti-VP6 conjugated to 6 nm gold **(d and e)** followed by rabbit mAb anti-CCT2 **(b and d)** or rabbit polyclonal anti-CCT2 **(c and e)** both conjugated to 12 nm gold. The white dashed open boxes correspond to enlarged indicated images. Red arrowheads and white arrows point to the localization of CCT2 and NSP5 or VP6 surrounding DLPs. The scale bar is 200 nm.

**Figure S3. Characterization of TRICi in MA104 cells**. **a)** Schematic representation of MA104 cells synchronized in the G1/S phase using double thymidine blocking. Immediately after thymidine release, the cells were untreated or treated with TRICi at 1.25 mM and 2.5 mM. At 7.5 h post-release, cells were harvested and examined for the percentage of cells in mitosis using an anti-histone H3 phosphorylated serine 328 (Alexa 647-H3SP) and expression of Cdc20. **b)** Flow cytometry diagram to characterize the percentage of cells in mitosis. **c)** Representative scatter plots for mitosis detection (upper panel) and histogram plots for DNA content (lower panel) of MA104 cells untreated or treated with TRICi at the indicated concentrations. The percentage of cells in mitosis is indicated in the top right corner of each scatter plot. In addition, the G_1/0_ and G_2_ cell cycle phases are indicated. **d)** Plot for the relative ratio of mitotic cells after treatment with TRICi. The data correspond to the mean ± SD of three independent experiments. Two-way ANOVA, (***), p<0.0001. **e)** Immunofluorescence images for the expression of Cdc20 (anti-Cdc20, red) of thymidine synchronized MA104 cells followed by treatment without or with TRICi at the indicated concentrations immediately after release. Nuclei were stained with DAPI (blue). The yellow arrows point to Cdc20-positive cells. The scale bar is 10 µm. **f)** Plot for cell viability of non-infected and RV-infected MA104 cells untreated or treated with TRICi at 1.25 or 2.5 mM. The chemical compound was added at 1 hpi, and samples were collected up to 12 hpi. The cell viability was determined by the release of lactate dehydrogenase. The data represent the mean ± SD of three independent experiments. Positive control corresponds to cells lysed in 0.1 % Triton X-100 buffer.

**Figure S4. TRIC inhibition hampers viroplasm formation of RV strains OSU, SA11, and RRV and silencing CCT3 and CCT2 TRiC subunits decreases virus progeny.** OSU-, SA11-, or RRV-infected (MOI, 25 VFU/ml) MA104 cells untreated (NT) and treated with 1.25 mM or 2.5 mM TRICi added at 1 hpi. **a)** Immunofluorescence images of cells fixed at 6 hpi and immunostained to detect viroplasms (anti-NSP5, green). Nuclei were stained with DAPI (blue). The scale bar is 10 µm. **b)** Immunoblotting of cell extract harvested at 6 hpi. The membrane was incubated with specific antibodies to detect the indicated proteins. GAPDH was used as a loading control. Immunoblotting of cellular extract silenced with siCCT3 **(c)** and siCCT2 **(d)**. Scramble siRNA (scrbl) is included. GAPDH was used as the loading control. Plot for virus progeny of OSU-infected MA104 cells silenced with scrambled siRNA, siCCT3 **(e),** or siCCT2 **(f)**. The data represent the mean ± SD of three independent experiments. Student's t-test; (*), p<0.05.

**Figure S5. TRICi effect is reversible over viroplasm formation**. **a)** Schematic timeline for the addition and washout of TRICi in RV-infected cells. At 6 hpi, all the samples were analyzed to detect viroplasms and the expression of diverse RV proteins. **b)** Immunofluorescence images of RV-infected cells (MOI, 25 VFU/cell) treated with TRICi at indicated periods. At 6 hpi, cells were fixed and immunostained to detect viroplasms (anti-NSP5, green). Nuclei were stained with DAPI (blue). The scale bar is 10 µm. **c)** Plot for quantifying the number of viroplasms after recovery from TRICi as described in **(a)**. Plots for the distribution of small (< 0.162 µm^2^) **(d)** and large (> 0.162 µm^2^) **(e)** viroplasms after recovery from TRICi. Data represent the mean ± SD. n> 50 cells. The data represent the mean ± SD. n>50 cells per point; Welch's two-way ANOVA compared to untreated condition where (**), p<0.001; (***), p<0.0001 and (****), p<0.00001. **f)** Immunoblotting of RV-infected cell extracts after recovery at the indicated treatment periods with TRICi. The membranes were incubated with the indicated specific antibodies. GAPDH was used as a loading control.

**Figure S6.** ***In vivo* NSP5 hyperphosphorylation assay in presence of TRICi and localization of TRiC components in VLSs. a)** *In vivo* phosphorylation assay of extracts from cells expressing NSP5 alone or together with VP2 or NSP2 with or without 2.5 mM TRICi. The membrane was incubated with the indicated antibodies. The red brackets indicate NSP5 hyperphosphorylation. GAPDH corresponds to loading control. **b)** Immunofluorescence images of VLSs composed of NSP5 with the indicated RV proteins. At 16 hpt, the cells were immunostained for detection of VLS (anti-NSP5, red), TRiC subunit CCT3 (anti-CCT3, green), and V5-VP1 (anti-V5, cyan). The nuclei were stained with DAPI (blue). A merged image is shown in the right column. The scale bar is 10 µm**.** **c)** Immunofluorescence images of VLS composed either NSP5 with NSP2 (left panel) or NSP5 with VP2 (right panel). At 16 hpt, the cells were fixed and immunostained for the detection of VLS (anti-NSP5, Alexa 488, green) and the TRiC components CCT1 (top row; anti-CCT1, Alexa 647, cyan) or CCT2 (bottom row; anti-CCT2, Alexa 674, cyan). Nuclei were stained with DAPI (blue). Scale bar is 10 µm.

**Figure S7. Analysis of OSU subviral particles isolated from infected cell extract untreated or treated with 2.5 mM TRICi.** a**)** Standardization method for detecting RV dsRNA genome segments using Agilent 2200 TapeStation genomic with DNA Screen tape. Plots corresponding to the chromatogram of the standard ladder (top panel) and OSU dsRNA genome segments extract (bottom panel). The observed molecular weight migration for each peak is indicated. **b)** Migration pattern of the ladder and OSU dsRNA genome segments extracted in the DNA screen tape. **c)** Migration pattern of OSU dsRNA genome segments extracted in SDS-PAGE followed by staining with Gel Red. **d)** Table comparing the predicted and observed migration pattern of each OSU dsRNA genome segment in DNA screen tape in TapeStation.

**Supplementary Tables**

**Table S1**. Reverse transcriptase adaptors used for Oxford Nanopore Technology direct RNA sequencing of RV positive and negative single-stranded RNAs

**Table S2.** Summary of LC-MS/MS of bands extracted from CBS lanes of fraction 1 (DMSO) and fraction 4 (TRICi) of CsCl gradient and Halotag-HA-VP2 extracts migrated in native gel

**Supplementary Materials and Methods**

**siRNA reverse transfection and infection.** siRNA reverse transfections were performed using Lipofectamine RNAi MAX Transfection Reagent following manufacturer instructions. For transfection in a 24-multiwell tissue culture plate, 1.2 µl siRNA 5µM were diluted to 100 µl with Opti-MEM™ (Gibco™, ThermoFisher) plus 1 µl of transfection reagent were added to one well and incubated for 20 min at room temperature. Then, 2x10^4^ MA104 cells diluted in 500 µl DMEM supplemented with 10%FCS were added on the top of the transfection. The final concentration reached by siRNA is 10 nM. At 48 hpt, cells were RV-infected at an MOI of 25 VFU per cell, as described previously (1). The scramble siRNA corresponds to Control siRNA-A (sc-37007; Santa Cruz Biotechnology, Inc). The siCCT3 corresponds to the following equimolar mix of RNA sequences: CCT3_5 siRNA: 5’-CAGACTGACATTGAGATTACA-3'; CCT3_9 siRNA: 5’-AGCGGCCAAGTCCA TGATCGA-3'; CCT3_8 siRNA: 5’-ATCCACGTATGCGGCGCTATA-3'; CCT3_7: 5'- CTTGCGTGGAGTCATGATTAA-3' were purchased at Sigma-Aldrich.

**Cell synchronization and flow cytometry.** MA104 cells were synchronized by double thymidine blocking, as described previously by Gluck et al., 2017 (2). Cells were released by washing twice with PBS and adding 3 ml of 10% FCS-DMEM without or with TRICi at the indicated concentrations. At 7.5 h post-release, cells were harvested by washing twice with PBS, detached with 0.5 ml of 0.5 % Trypsin-EDTA (ThermoFisher), collected in 15 ml tubes with 3 ml cDMEM, and centrifuged for 2 min at 1500 rpm. The cell pellet was resuspended in 1 ml PBS and mixed gently with 2.5 ml ethanol 100%. The samples were stored at -20ºC overnight. Then, samples were centrifuged for 5 min at 1500 rpm at 4ºC, and the pellet was washed once with 1 ml of PBS, followed by a second wash with 1 ml of staining buffer (1% Fetal bovine serum in PBS). The cell pellet was resuspended in 100 µl of Alexa 647-anti-histone H3-Phosphorylated (Ser38) antibody (clone HTA28) (BioLegend) diluted 1:20 in staining buffer and incubated for 20 min at room temperature. Cells were centrifuged for 5 min at 1500 rpm and 4ºC, and the pellet was resuspended in 500 µl of propidium iodide solution (0.05 % Triton X-100, 0.1 mg/ml RNAse A, 50 µg/ml PI in PBS), incubated for 40 min at 37ºC in the dark and filtered using a cell strainer snap-cap tube (BD Falcon™). Samples were immediately acquired using a CytoFLEX S flow cytometer (Beckman Coulter). Thus, 25'000 events were acquired exciting at both 488 nm with a blue laser and filter band of 610/20 and 638 nm with a red laser and filter band 660/10. Data were analyzed and processed using Kaluza Analysis Software (Beckman Coulter). For the detection of Cdc-20 by immunofluorescence, a coverslip was included in the second blocking of thymidine. At the indicated time post-release, the cells in slides were fixed in 2% paraformaldehyde for 10 min at room temperature, permeabilized with 0.1% Triton X-100 in PBS for 5 min at room temperature and blocked with 1% BSA-PBS for 20 min at room temperature. Expression of Cdc20 was detected by immunostaining with a rabbit anti-Cdc20 followed by a secondary antibody anti-rabbit conjugated to Alexa 594. Nuclei were stained with 0.01 mg/ml DAPI in PBS. Coverslips were mounted in ProLong™ Gold Antifade Mountant (ThermoFisher). Images were acquired at CSLM SP8 inverse (Leica) using a 63X HCPL APO CS2 lens and analyzed using ImageJ2 version: 2.3.0/1.53q.

**In vivo hyperphosphorylation assay.** Transfection and cellular extract preparation is described in detail by Buttafuoco et al., 2020 (3). At 3.5 hpt, media was replaced by complete media containing 2.5 mM TRICi. Samples were analyzed by immunoblotting as described previously (4).

**LC-MS/MS**. The gel bands were cut into small pieces and washed twice in 100 μl wash solution (100 mM NH4HCO3, 50% (v/v) acetonitrile) and once in acetonitrile. The supernatant was discarded after each wash step. The proteins were digested using 10 µl of Sequencing Grade Trypsin (5 ng/µl in 10 mM Tris, 2 mM CaCl_2_, pH 8.2, Promega) and 30 µl of digestion buffer (10 mM Tris, 2 mM CaCl_2_, pH 8.2). The digestion was done in a microwave instrument (Discover System, CEM) for 30 min at 5 W and 60 °C. The supernatants were collected, and the peptides were extracted from the gel pieces using 150 µl 0.1% trifluoroacetic acid and 50% acetonitrile (15 min in an ultrasonic bath). The supernatants were combined, and the samples were dried entirely and resolubilized in 20 µl of MS sample buffer (3% acetonitrile, 0.1% formic acid). LC-MS/MS analysis was performed on a Q Exactive mass spectrometer (Thermo Scientific) equipped with a Digital PicoView source (New Objective) and coupled to an M-Class UPLC (Waters). The solvent composition at the two channels was 0.1% formic acid for channel A and 0.1% formic acid 99.9% acetonitrile for channel B. For each sample, 2 µl of peptides were loaded on a commercial ACQUITY UPLC M-Class Symmetry C18 Trap Column (100Å, 5 µm, 180 µm x 20 mm, Waters) connected to an ACQUITY UPLC M-Class HSS T3 Column (100Å, 1.8 µm, 75 µm X 250 mm, Waters). The peptides were eluted at a flow rate of 300 nL/min. After a 3-minute initial hold at 5% B, a gradient from 5 to 45 % B in 42 min and 35 to 40% B in an additional 5 min was applied.

The samples were measured with blank injections between. The mass spectrometer was operated in data-dependent mode (DDA) using Xcalibur and heated capillary temperature at 275 °C. Full-scan MS spectra (350−1500 m/z) were acquired at a resolution of 70'000 at 200 m/z after accumulation to a target value of 3'000'000, followed by HCD (higher-energy collision dissociation) fragmentation on the twelve most intense signals per cycle. Ions were isolated with a 1.2 m/z isolation window and fragmented by higher-energy collisional dissociation (HCD) using a normalized collision energy of 25 %. HCD spectra were acquired at a resolution of 35'000 and a maximum injection time of 120 ms. The automatic gain control (AGC) was set to 100'000 ions. The charge state screening was enabled, and single and unassigned charge states were rejected. Only precursors with intensity above 25,000 were selected for MS/MS. Precursor masses previously selected for MS/MS measurement were excluded from further selection for 10 s, and the exclusion window tolerance was set at 10 ppm. The samples were acquired using internal lock mass calibration on m/z 371.1010 and 445.1200. The mass spectrometry proteomics data were handled using the local laboratory information management system (LIMS) (5).

**Supplementary References**

1. Knowlton JJ, Fernández de Castro I, Ashbrook AW, Gestaut DR, Zamora PF, Bauer JA, Forrest JC, Frydman J, Risco C, Dermody TS. 2018. The TRiC chaperonin controls reovirus replication through outer-capsid folding. Nat Microbiol 3:481-493.

2. Glück S, Buttafuoco A, Meier AF, Arnoldi F, Vogt B, Schraner EM, Ackermann M, Eichwald C. 2017. Rotavirus replication is correlated with S/G2 interphase arrest of the host cell cycle. PLoS One 12:e0179607.

3. Buttafuoco A, Michaelsen K, Tobler K, Ackermann M, Fraefel C, Eichwald C. 2020. Conserved rotavirus NSP5 and VP2 domains interact and affect viroplasm. J Virol 94.

4. Eichwald C, Arnoldi F, Laimbacher AS, Schraner EM, Fraefel C, Wild P, Burrone OR, Ackermann M. 2012. Rotavirus viroplasm fusion and perinuclear localization are dynamic processes requiring stabilized microtubules. PLoS ONE 7.

5. Türker C, Akal F, Schlapbach R. 2011. Life sciences data and application integration with B-fabric. J Integr Bioinform 8:159.
