# Supplementary material for: The recruitment of TRiC chaperonin in rotavirus viroplasms correlates with virus replication
Source: mBio. 2024 Mar 12;15(4):e00499-24. doi: 10.1128/mbio.00499-24 (PMC11005421; doi:10.1128/mbio.00499-24)
Supplement: Table S1 — Reverse transcriptase adaptors used for Oxford Nanopore Technology direct RNA sequencing of RV positive and negative single-stranded RNAs. [file mbio.00499-24-s0009.docx]

| **Table S1.** | | Synthetic primers Reverse transcriptase adaptors used for Oxford Nanopore Technology direct RNA sequencing of RV positive and negative single stranded RNAs | |
| --- | --- | --- | --- |
| **name** | **oligonucleotide annealing**  **positive strand** | | **oligonucleotide annealing**  **negative strand** |
| gs 1_  (VP1) | 5’-*gaggcgagcggtcaattttcctaaga*  *gcaagaagaagcc***ggtcacatct**-3’ | | 5’-*gaggcgagcggtcaattttcctaaga*  *gcaagaagaagcc***ggctattaaa**-3’ |
| gs2_  (VP2) | 5’-*gaggcgagcggtcaattttcctaaga gcaagaagaagcc***ggtcatatct**-3’ | | 5’-*gaggcgagcggtcaattttcctaaga*  *gcaagaagaagcc***ggctattaaa**-3’ |
| gs3_  (VP3) | 5’-*gaggcgagcggtcaattttcctaaga gcaagaagaagcc***ggtcacatcg**-3’ | | 5’*-gaggcgagcggtcaattttcctaaga*  *gcaagaagaagcc***ggctattaaa**-3’ |
| gs4_  (VP4) | 5’-*gaggcgagcggtcaattttcctaaga gcaagaagaagcc***ggtcacaacc**-3’ | | 5’-*gaggcgagcggtcaattttcctaaga*  *gcaagaagaagcc***ggctataaaa**-3’ |
| gs5_  (NSP1) | 5’-*gaggcgagcggtcaattttcctaaga gcaagaagaagcc***ggtcacattt**-3’ | | 5’-*gaggcgagcggtcaattttcctaaga*  *gcaagaagaagcc***ggcttttttt**-3’ |
| gs6_  (VP6) | 5’-*gaggcgagcggtcaattttcctaaga gcaagaagaagcc***ggtcacatcc**-3’ | | 5’-*gaggcgagcggtcaattttcctaaga gcaagaagaagcc***ggctttaaaa**-3’ |
| gs7_  (NSP3) | 5’-*gaggcgagcggtcaattttcctaaga gcaagaagaagcc***ggtcacataa**-3’ | | 5’-*gaggcgagcggtcaattttcctaaga gcaagaagaagcc***ggcttttaat**-3’ |
| gs8_  (NSP2) | 5’-*gaggcgagcggtcaattttcctaaga gcaagaagaagcc***ggtcacataa**-3’ | | 5’-*gaggcgagcggtcaattttcctaaga*  *gcaagaagaagcc***ggcttttaaa**-3’ |
| gs9_  (VP7) | 5’-*gaggcgagcggtcaattttcctaaga gcaagaagaagcc***ggtcacatca**-3’ | | 5’-*gaggcgagcggtcaattttcctaaga*  *gcaagaagaagcc***ggctttaaaa**-3’ |
| gs10_  (NSP4) | 5’-*gaggcgagcggtcaattttcctaaga gca agaagaagcc***ggtcacacta**-3’ | | 5’-*gaggcgagcggtcaattttcctaaga*  *gcaagaagaagcc***ggcttttaaa**-3’ |
| gs11_  (NSP5) | 5’-*gaggcgagcggtcaattttcctaaga gcaagaagaagcc***ggtcacaaaa**-3’ | | 5’-*gaggcgagcggtcaattttcctaaga*  *gcaagaagaagcc***gcgctacagt** |
| GAPDH_  MA104 | 5’-*gaggcgagcggtcaattttcctaa gagcaagaagaagcc***ggggatttta**-3’ | | 5’-*gaggcgagcggtcaattttcctaaga*  *gcaagaagaagcc***ccaaggtcat**-3’ |
| RT-Adapter-oligoA | 5’-[5PHOS]-ggcttcttcttgct cttaggtagtaggttc-3’ | |  |

1. Specific RTA sequence is labeled in cursive
2. The 3'ends of the positive and negative RNA strands of each rotavirus genome segments sequence are labeled in bold.
