# Supplementary material for: The recruitment of TRiC chaperonin in rotavirus viroplasms correlates with virus replication
Source: mBio. 2024 Mar 12;15(4):e00499-24. doi: 10.1128/mbio.00499-24 (PMC11005421; doi:10.1128/mbio.00499-24)
Supplement: Table S2 — Summary of LC-MS/MS of bands extracted from CBS lanes of fraction 1 (DMSO) and fraction 4 (TRICi) of CsCl gradient and Halotag-HA-VP2 extracts migrated in native gel. [file mbio.00499-24-s0010.docx]

| **treatment** | **CBS^a^** | **expected protein** | **VP1^b,d^** | **VP2^b,d^** | **VP3^b,d^** | **VP4^b,d^** | **VP6^b,d^** | **VP7^b,d^** |
| --- | --- | --- | --- | --- | --- | --- | --- | --- |
| DMSO | B1 | (VP1) | **64%, 77** | 44%, 35 | 0 | 26%,17 | 30%, 9 | 11%, 3 |
|  | B2 | (VP2,VP3,VP4) | 8%, 8 | **77%, 117** | **16%, 11** | **64%, 56** | 21%, 10 | 10%, 2 |
|  | B3 | (VP6) | 0 | 33%, 21 | 0 | 23%,11 | **97%, 76** | 14%, 3 |
|  | B4 | (VP7) | 0 | 6%, 3 | 0 | 16%,10 | 55%, 13 | **57%, 26** |
| TRICi | B1 | (VP1) | **16%, 18** | 6%, 5 | 0 | 2%, 2 | 20%, 3 | 0 |
|  | B2 | (VP2,VP3,VP4) | 0 | **50%,40** | 2%, 1 | **24%, 14** | 23%, 4 | 0 |
|  | B3 | (VP6) | 0 | 0 | 0 | 0 | **33%, 9** | 0 |
|  | B4 | (VP7) | 0 | 0 | 0 | 1%, 1 | 6%, 1 | **42%, 11** |
| **treatment** | **CBS^c^** | **Halotag-HA-VP2^b,d^** | | | | | | |
| DMSO | B5 | **34%, 38** | | | | | | |
|  | B6 | **21%, 19** | | | | | | |
| TRICi | B5 | **22%, 22** | | | | | | |
|  | B6 | **18%, 20** | | | | | | |

**Table S2.** Summary of LC-MS/MS of bands extracted from CBS lanes of fraction 1 (DMSO) and fraction 4 (TRICi) of CsCl gradient and Halotag-HA-VP2 extracts migrated in native gel.

^a^ CBS bands corresponding to the following MW sections: B1, 100-150 kDa; B2, 75-100 kDa; B3, above 37 kDa; and B4,below 37 kDa. The bands corresponded to the migration of VP1 (B1); VP2, VP3 and VP4 (B2), VP6 (B3) and VP7 (B4) of fraction 3 (DMSO).

^b^ % sequence coverage, number peptides.

^c^ B5 and B6 correspond to the migration of bands excised for high and low order, respectively.

^d^ Bold numbers indicate >95% probability of protein present
